# Survival, Growth, and Reproduction Responses in a Three-Generation Exposure of the Zebrafish (*Danio rerio*) to Perfluorooctane Sulfonate

**DOI:** 10.1002/etc.5770

**Published:** 2023-11-29

**Authors:** Kurt A. Gust, J. Erik Mylroie, Ashley N. Kimble, Mitchell S. Wilbanks, Catherine S. C. Steward, Kacy A. Chapman, Kathleen M. Jensen, Alan J. Kennedy, Paige M. Krupa, Scott A. Waisner, Zacharias Pandelides, Natalia Garcia-Reyero, Russell J. Erickson, Gerald T. Ankley, Jason Conder, David W. Moore

**Affiliations:** a Environmental Laboratory, Engineer Research and Development Center, US Army, Vicksburg, Mississippi, USA; b Bennett Aerospace, Cary, North Carolina, USA; c Oak Ridge Institute for Science and Education, Oak Ridge, Tennessee, USA; d Great Lakes Toxicology and Ecology Division, US Environmental Protection Agency, Duluth, Minnesota, USA; e Geosyntec Consultants, Costa Mesa, California, USA

**Keywords:** Zebrafish, Perfluorooctane sulfonic acid (PFOS), Multigenerational exposure, Growth, Reproduction

## Abstract

A prior multigenerational perfluorooctane sulfonic acid (PFOS) exposure investigation in zebrafish reported adverse effects at 0.734 μg/L, among the lowest aquatic effect levels for PFOS reported to date. The present three-generation PFOS exposure quantified survival, growth, reproduction, and vitellogenin (VTG; egg yolk protein) responses in zebrafish, incorporating experimental design and procedural improvements relative to the earlier study. Exposures targeting 0.1, 0.6, 3.2, 20, and 100 μg/L in parental (P) and first filial (F1) generations lasted for 180 days post fertilization (dpf) and the second filial generation (F2) through 16 dpf. Survival decreased significantly in P and F2 generation exposures, but not in F1, at the highest PFOS treatment (100 μg/L nominal, 94–205 μg/L, measured). Significant adverse effects on body weight and length were infrequent, of low magnitude, and occurred predominantly at the highest exposure treatment. Finally, PFOS had no significant effects on P or F1 egg production and survival or whole-body VTG levels in P or F1 male fish. Overall, the predominance and magnitude of adverse PFOS effects at <1 μg/L reported in prior research were largely nonrepeatable in the present study. In contrast, the present study indicated a threshold for ecologically relevant adverse effects in zebrafish at 117 μg/L (SE 8 μg/L, *n* = 10) for survival and 47 μg/L (SE 11 μg/L, *n* = 19) for all statistically significant negative effects observed.

## INTRODUCTION

The emergence of per- and polyfluoroalkyl substances (PFAS) as a global issue (Kurwadkar et al., 2022; De Silva et al., 2021; Sunderland et al., 2019) necessitates accurate characterization of harmful biological effects to minimize uncertainty in establishing both human and ecological health risks. The identification of organisms susceptible to PFAS is a critical need for ecological risk characterization (Ankley et al., 2021). Furthermore, species concordance, especially for the most sensitive and impactful health outcomes for PFAS, is critical for advancing our knowledge of risks and strategies for human health protection (Fenton et al., 2021). The availability of high-quality, peer-reviewed publications characterizing effect concentrations remains a central requirement for establishing defensible evidence-based regulatory thresholds (Ankley et al., 2021; Harris et al., 2014). Although scientific peer review establishes some degree of quality assurance for published experimental outcomes, further scrutiny is needed of the quality of published results for possible use in regulatory applications (US Environmental Protection Agency [USEPA], 2003, 2011).

The present study investigated the effects of aquatic exposures to perfluorooctanesulfonic acid (PFOS), one of the most prevalent and broadly distributed PFAS (Paul et al., 2009; Podder et al., 2021). Perfluorooctanesulfonic acid also exhibits one of the highest relative potencies among the diverse array of PFAS molecular structures (Bil et al., 2021). The aquatic toxicity of PFOS was investigated in zebrafish, a well-established environmental toxicology model (Scholz et al., 2008) and US National Institute of Health-endorsed human health model species (Dooley & Zon, 2000). Zebrafish studies have already proved to be important for PFAS risk characterization for both ecological and human health protection applications (Gaballah et al., 2020; Han et al., 2021; Tal & Vogs, 2021). The present study was initiated to reconcile questions and establish the reproducibility of results from a previously published study of multigenerational zebrafish PFOS exposures (of 0.734, 106.9, and 267.6 μg/L [measured]; Keiter et al., 2012). The study reported reduced growth (length and weight) responses at 0.734 μg/L at multiple chronic exposure time points (30, 90, and 180 days post fertilization [dpf]) in both parental (P) and first filial (F1) generations (Keiter et al., 2012). This effect concentration deviated by almost 2 orders of magnitude from the next most sensitive observation for continuous chronic PFOS exposure to zebrafish (from embryo through 152 dpf), which reported a lowest-observed-effect concentration (LOEC) of 50 μg/L (nominal) for reproduction (Wang et al., 2011).

Species sensitivity distributions typically used by regulatory bodies to establish chemical toxicity thresholds can be highly influenced by the lowest reported effect concentrations (Belanger et al., 2017). The Environmental Protection Authority of Victoria, Australia, applied the 0.734 μg/L effect concentration reported in Keiter et al. (2012) in the derivation of its water quality guidelines for PFOS (Department of Environment and Energy of the Australian Government, 2016), whereas the USEPA (2022) did not include this effect in its calculation of draft ambient water quality criteria for PFOS, citing a lack of concentration–response relationships due to limitations of the study design (i.e., a small number of concentrations and low replication).

The scope of the Keiter et al. (2012) study was ambitious and provided results that are typically lacking in toxicological research, such as continuous extended long-term and multi-generational exposures. The potential influence of their results on regulatory thresholds motivated our reproduction of the multigeneration investigation, but with a more robust experimental design. In the present study, we increased the number of PFOS treatments (from three to five, plus control) with the exposure concentrations being more narrowly spaced and evenly distributed and a log scale to support robust dose–response relationships. In addition, the present study incorporated increased replication (from two to five replicates/treatment), and care was taken to maintain the independence of the replicates throughout the multigenerational exposure. In addition, statistical model selection was thoroughly explored to best fit the data structure, test objectives, and meet underlying statistical assumptions. Analytical confirmation of PFOS exposure concentrations was also greatly expanded and included multiple rounds of external validation provided by third-party analytical laboratories. We used the standard endpoints of survival, growth, and reproduction as well as vitellogenin (VTG) levels in males as an indicator of potential endocrine disruption (Arcand-Hoy & Benson, 1998).

## METHODS

### Water treatment system

**FIGURE 1:**
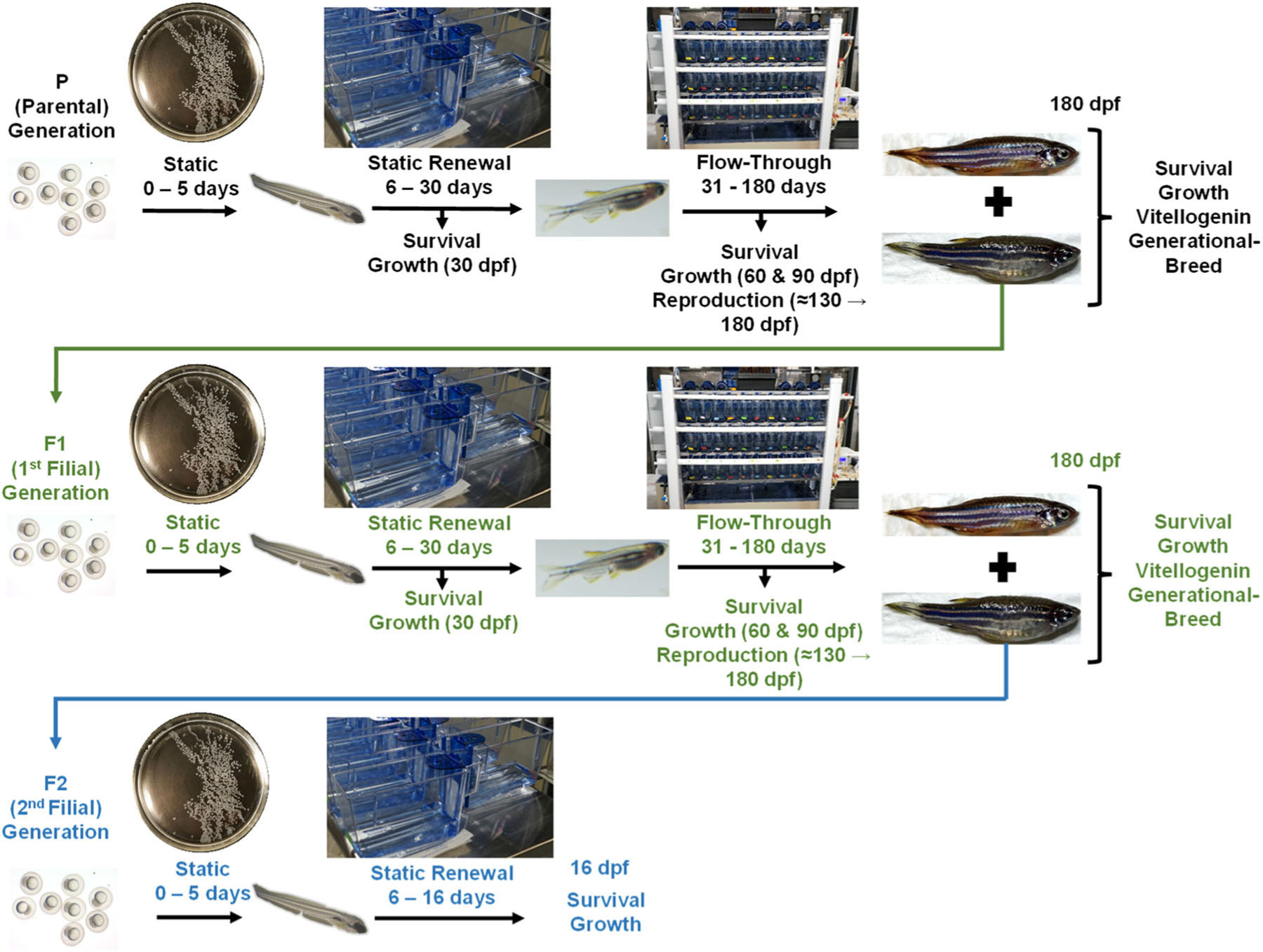
Graphical flow chart of the multigenerational exposure of zebrafish to perfluorooctanesulfonic acid (PFOS) with associated exposure methods, exposure times (dpf = days post fertilization), and experimental endpoints that were measured.

Water used for all exposures was Vicksburg (MS, USA) municipal water first treated by reverse osmosis followed by serial passage through two canisters of Purofine^®^ PFA694 ionic resin (Purolite) to remove any residual contaminants (including fluorinated chemicals) from the water (Supporting Information, S1, Figure 1). Hereafter, the term “control water” refers to the water processed using this treatment. The methods used for exposure water clean-up post exposure are provided in the Supporting Information, S1, Methods.

### Experimental design

The experimental design for the multigenerational PFOS test included control and 0.1, 0.6, 3.2, 20, and 100 μg/L PFOS treatments administered as continuous exposures through 180 dpf in the parental (P) generation, 180 dpf in the F1 generation, and 16 dpf in the F2 generation. The PFOS concentrations were selected to bracket the exposures investigated in Keiter et al. (2012), focusing on the 0.6 μg/L PFOS treatment and extending additional concentrations above and below on a log-based scale. To support the development of a robust experimental design, a priori sample size and power analyses were conducted across survival, growth, and reproduction endpoints to ensure a minimum statistical power of 0.8 for the parametric statistical tests described in the *Statistical analyses* section following. Descriptions of sample size and power analyses are provided in the Supporting Information, S1, Methods, and results are presented in the Supporting Information, S1, Table S1. Ultimately, the minimum sample size necessary to meet the power and test sensitivity requirements for a parametric analysis of variance (ANOVA) with six treatment levels (control and the five PFOS exposure concentrations) measuring zebrafish survival, growth, and reproduction endpoints was five replicates (*n* = 5). Therefore, experimental exposures consisted of five replicate chambers starting with 50 fish/chamber. Fish from each replicate chamber were kept separate from the other chambers through the entire duration of the multigenerational exposure, to maintain independence. All animal testing was conducted using methods and protocols approved by the US Army Engineer Research and Development Center’s Institutional Animal Care and Use Committee (IACUC Protocol # EL-6008-2020-1).

### PFOS exposure preparation

Heptadecafluorooctanesulfonic acid potassium salt (PFOS; CAS No. 2795-39-3; >98% purity; Product #: 77282, Lot #: BCCC4690) was obtained from Sigma-Aldrich. An 8-L stock solution of 100 mg PFOS/L was prepared in ultrapure water produced with a Milli-Q^®^ water purification system (MilliporeSigma), and stocks were apportioned into aliquots and stored at −20 °C. The stock solution was analyzed in triplicate using the analytical chemistry methods described in the section *Analytical chemistry methods*, and all exposure solutions were prepared based on the measured concentration of the PFOS stock solution. For the embryo static exposures, the exposure solutions of 0.1, 0.6, 3.2, 20, and 100 μg PFOS/L were prepared in zebrafish embryo media (E2) (Varga, 2016). The larval exposure solutions were prepared at the same concentrations but used the control water supplemented with Instant Ocean (Spectrum Brands) to a salinity of 1.0 parts/1000 (ppt) to support live rotifer feeds. (Feeding methods are described in the later section, *Multigenerational PFOS exposures in zebrafish*.) As the precursors to the nominal target PFOS concentrations administered in the zebrafish exposures, a 100-mg/L PFOS stock solution was first diluted into PFOS stocks that were 10× concentrates prepared in 10 to 20-L volumes of control water. Once prepared, these solutions were used to fill 20-L opaque carboys, which supplied the fish exposure system, to achieve the targeted nominal exposure concentrations. The PFOS solutions were constantly stirred using glass-coated stir bars and stored at the ambient temperature of the exposure room (≈27 °C).

### Zebrafish embryo production for PFOS exposure

The wild-type AB strain adult zebrafish (Zebrafish International Resource Center) used to generate the parental (P) generation was maintained as described in Mylroie et al. (2021). The P generation was bred in an iSpawn (Tecniplast) breeding chamber using 58 males and 30 females as parents; spawning occurred for 30 min initiated by the start of the daily light cycle. After spawning was completed, the embryos were collected and counted, allowed to develop for approximately 1 h, and then washed and surface-sanitized using a dilute bleach solution following a protocol from Varga and Murray (2016) whereby the chorion was left intact.

### Multigenerational PFOS exposures in zebrafish

A conceptual overview of the complete multigenerational exposure is provided in Figure 1. The following text summarizes the general methods used for the PFOS exposures; more detailed experimental methods are provided in the Supporting Information, S1, Methods where specifically denoted. Exposure to the experimental treatments was initiated at approximately 7 hours post fertilization (hpf) in 75-mm polypropylene Petri plates (Eisco Scientific) containing 23 mL of each PFOS or control treatment. The zebrafish embryos were then incubated in an environmental chamber (Model# TE055-AA-LT; Darwin Chamber^®^) at 28.5 °C with a 14:10-h day:night cycle, which continued through 5 dpf. At 5 dpf, 50 zebrafish were transferred to replicate 3-L polypropylene exposure chambers, which contained approximately 800 mL of the PFOS treatment solutions (or control) prepared in E2 media. Feeding was initiated by providing a rotifer suspension and GEMMA Micro 75 (Skretting USA) as food. The methods of rotifer preparation and zebrafish feeding are provided in the Supporting Information, S1, Methods. The exposures were maintained with static renewal of exposure solutions from 5 to 30 dpf with all replicates receiving an 80% water exchange of 1 ppt Instant Ocean–supplemented control water twice daily; exposure volumes were gradually increased starting at 15 dpf by adding an extra 100 mL of volume during each water exchange for 6 days, resulting in a final tank volume of 2 L. The 2-L volume was then maintained through 30 dpf.

At 30 dpf, the zebrafish were transferred to a ZebTEC Stand-Alone Toxicology Rack (Tecniplast) flow-through exposure system (Supporting Information, S1, Figures S2 and S3), after which the rotifer feeding ceased, and GEMMA Micro 75 feeding was increased to three times/day, which was then replaced by feeding with 40 mg GEMMA Micro 150, twice/day at 50 through 111 dpf/replicate. Details of the flow-through zebrafish exposure system setup and operation are provided in the Supporting Information, S1, Methods. As an overview, the system housed zebrafish in individually aerated replicate polycarbonate chambers containing 2.8 L of experimental treatment water/chamber, which was exchanged three times daily. In conjunction with the system water exchanges, peristaltic pumps (Fisherbrand™ FH100M MultiChannel; Fisher Scientific) were calibrated to deliver PFOS treatment solutions or control water simultaneously with the water exchange to maintain PFOS exposure concentrations (Supporting Information, S1, Figures S2 and S3); mixing vessels were included within each chamber to eliminate PFOS concentration spikes after delivery (Supporting Information, S1, Figure S4). Temperature, conductivity, and pH were automatically monitored and maintained by the water quality monitoring system on-board the exposure system. All exposure chambers were preconditioned for 14 days by addition of the respective PFOS exposure treatment concentration before the addition of fish.

At 111 dpf, the fish within each replicate chamber were reduced to 15 males and 15 females to establish an even sex ratio in preparation for reproductive assays; this ratio was maintained through 180 dpf. At this time, the zebrafish were fed GEMMA Micro 500 fish food twice a day at a ration of 3.8 mg of food/fish using either preweighed food aliquots or a calibrated hand-held granular feeder (Pentair). To initiate the F1 generation, all fish within each replicate chamber from the P generation were bred at 180 dpf, and separation was carefully maintained among all replicates. The same process was used to breed the F1 fish to produce the F2 generation. For each generation, a back-up set of embryos was produced at 185 dpf to serve as a brood stock to insure against experimental accidents or failures. Complete sets of detailed breeding methods for primary and back-up embryo sets are provided in the Supporting Information, S1, Methods. For the F1 generation, the back-up set of embryos moved forward through the generational exposure because the initial F1 embryo set failed to thrive due to poor quality of rotifers used as live food, which caused high mortality across all treatments including controls. The F2 generation proceeded with the primary F2 embryo set. After each breeding event, the embryos were processed and introduced to their respective PFOS exposure treatments using the methods just described. At the completion of each generational exposure, fish were euthanized by an overdose of buffered 4 g/L MS-222 (CAS no. 886-86-2; Product #: A5040; Sigma-Aldrich), and all fish were measured for growth metrics. Some of the fish were flash-frozen whole for whole body VTG analyses, and other fish were dissected, and the brains, livers, kidneys, and gonads were removed and preserved for potential future analyses. Details on growth measurements and VTG assays are described in the subsequent Methods sections or the Supporting Information, S1, Methods.

### Zebrafish survival

Zebrafish mortality was recorded by daily counts of dead fish found in each replicate. All surviving fish in each replicate were counted during growth measurements (described in the following section, *Zebrafish growth measurements*) at 30, 60, 90, and 180 dpf for the P and F1 generations and at 16 dpf for the F2 generation. Counts of surviving fish at 30 dpf compared against daily mortality counts indicated that some replicates did not contain exactly 50 fish at the start of each generational exposure due to counting errors. The mean starting numbers of fish for the P, F1, and F2 generational exposures were 49.2 (2.7 standard deviation [SD]), 51.6 (2.6 SD), and 49.7 (2.6 SD), respectively. All survival calculations were based on the counted number of fish present in each replicate at the beginning of each generational exposure where analyses were conducted to investigate PFOS effects on survival at 10, 15, 30, 60, 90, 111, and 180 dpf. The percentage of survival for the 180-dpf time point was calculated differently than the other time points due to the number of fish in each replicate at 111 dpf being reduced to achieve 15 males and 15 females. Given this reduction in the population size after 111 dpf, the basis for calculating the percentage of survival between 111 and 180 dpf was less than the prior time points. Therefore, the percentage of survival for the 180-dpf time point was calculated as the percentage of survival from 0 through 111 dpf multiplied by the percentage of survival from 111 through 180 dpf.

### Zebrafish growth measurements

Zebrafish body lengths and body weights were measured at selected time points in each generational exposure. Zebrafish body lengths were collected at 30- (or 34- for F1), 60-, 90-, and 180-dpf time points for both the P and F1 generations and at 16 dpf in the F2 exposure (methods, results, and interpretations for body length investigations are provided in the Supporting Information, S1, Materials, Results, and Discussion). Total body wet-weight measurements were taken at 60 and 90 dpf for the P and F1 generations in every replicate by collecting multiple pools of 8 to 12 fish into preweighed embryo collection baskets, blotting the basket briefly on a paper towel to remove excess water, and then weighing it on a balance (Practum^®^ Precision Balance, Product #: Practum313-1S; Sartorius Lab Instruments). Fish sex was not determined for the 60- and 90-dpf measurements. After weight measurements, the fish were immediately returned to the original replicate chamber on the exposure system for continued exposure within the experimental system. The 180-dpf weight measurements for both the P and F1 generations proceeded using euthanized fish that were pat-dried using a Kimwipe (Kimberly-Clark) and weighed on a balance, after which the sex of each fish was recorded. For F2 zebrafish weight measurements, fish were euthanized at 16 dpf, carefully blot-dried as just described and weighed in pools of approximately 16 fish on a balance.

### Zebrafish reproduction assays

Breeding trials to assess total egg production/female and egg survival after 24 h were carried out once weekly for 8 consecutive weeks in both the P and F1 generations. The breeding trials in the P and F1 generations began at 131 and 129 dpf, respectively. Each trial was conducted by impartially selecting five males and five females from each replicate, placing them in a 1.7-L Slope Breeding Tank (Tecniplast) filled with control water, where males and females were separated by a plastic divider the afternoon before the breeding trial. The next morning, at the beginning of the diurnal light cycle, the divider was removed, and the fish were allowed to spawn for approximately 45 min. Following the spawning period, the eggs produced in each breeding chamber were collected using an egg basket, and the total number of eggs was counted and recorded for each replicate. A maximum of 250 impartially selected eggs from each replicate were washed and surface sanitized using the methods described in the section *Zebrafish embryo production for PFOS exposure*, and then 48 impartially selected, noncoagulated eggs were placed in one 48-well plate/replicate, with each well containing one egg in 1 mL of E2 media. When fewer than 48 eggs were available, all the available noncoagulated eggs were plated and examined for survival. Replicates in which no eggs were produced were not included in the 24-h survival assessment. Plates were sealed with Parafilm M^®^ (Bemis) and placed in an incubator overnight at 28.5 °C at the same light cycle as throughout the study. Plates were assessed the next morning by counting all surviving eggs relative to the starting number of eggs.

### Whole-body VTG in male zebrafish

The effect of PFOS exposure on VTG (egg yolk protein precursor) concentrations in male zebrafish whole-body tissues was determined using an enzyme-linked immunosorbent assay (ELISA) targeting VTG (zebrafish VTG ELISA kit, Product #: V01008402-096; Biosense Laboratories). At 180 dpf, at least one male fish was collected from each of the five replicate exposure chambers for all experimental treatments, and an additional three males were collected impartially from individual replicates within each treatment, for a total of 48 fish assayed in each of the P and F1 generations. Two control female samples were also analyzed from the P and F1 generations to help confirm efficacy of the ELISA for detecting VTG.

The individual whole zebrafish were flash-frozen and then homogenized in a 7-mL conical homogenizer (Kimble^®^, Product #: 885300–0007; Kimble Chase) in ice-cold sample buffer at a ratio of 1 mg tissue/2 μL buffer. The sample buffer was comprised of a 1% bovine serum albumin (Product #: 03117332001; Roche Diagnostics) solution in phosphate-buffered saline (Product #: P5368, Lot #: SLCF6812; Sigma-Aldrich) with aprotinin (Product #: A6279, Lot #: SLCC9045; Sigma-Aldrich) added at a ratio of 2 trypsin inhibitor U/mL as per the Biosense zebrafish VTG ELISA kit protocol. The whole-body homogenate was transferred to a 5-mL tube, centrifuged at 4 °C at 20 000 *g* for 30 min, and then placed on ice. Vitellogenin concentrations were measured using a zebrafish VTG ELISA (Biosense Laboratories) kit according to the manufacturer’s protocol. Additional dilutions of the samples, beyond the recommended dilution series, were analyzed to ensure a sample result within the working range of the standard curve when necessary.

### Water quality

Standard water quality parameters including dissolved oxygen, temperature, conductivity, and pH were measured using a YSI Professional Plus multimeter (Xylem), were measured from a pooled sample of all replicates daily and total ammonia (Ammonia Nitrogen–Low Range; Product #: 3659-01-SC; LaMotte) measured once a week for all generations (Supporting Information, S1, Table 2). The mean conductivity for the static exposures was maintained higher than the target range for the flow-through exposure to improve the ability of the rotifers to stay in the water column and thus facilitate easier feeding by the zebrafish larvae. The conductivity means for the P and F1 flow-through portions of the exposures were marginally higher (2%) than the targeted range. This was due to a planned, slow decrease of conductivity to allow the zebrafish to acclimate to the target range once the live rotifer feeding had ceased. Methods for assessing alkalinity and total hardness are provided in the Supporting Information, S1, Methods. All water parameter measurements were within acceptable ranges for zebrafish housing conditions, as specified in Varga (2016) and the Organisation for Economic Co-operation and Development (2011) fish sexual development standard test.

### PFOS exposure confirmation

Perfluorooctanesulfonic acid was measured in exposure water samples taken every 1 to 2 weeks across the P, F1, and F2 generations through the complete duration of the multi-generation study. Greater numbers of water samples were taken during the first 6 weeks of exposure for the P and F1 generations, to closely maintain the intended nominal concentrations in the exposure system. Seven milliliters of water were collected from each treatment (control and 0.1, 0.6, 3.2, 20, and 100 μg PFOS/L) in 15-mL polypropylene centrifuge tubes at each sampling time, at which point two replicate exposure chambers/treatment were sampled. One exposure chamber replicate was measured repeatedly throughout the study, whereas the second exposure chamber replicate was impartially selected at each sampling period. Samples were stored at 4 to 6 °C in the dark for up to 14 days until in-house analysis was conducted.

### Analytical chemistry methods

The 7-mL water samples were diluted in the original collection vessel with 7 mL of methanol (MeOH). After the addition of MeOH, the samples were further diluted as needed to fall within the instrument’s linear range for PFOS. In the final dilution, an internal standard was present at 0.7 μg/L. Samples containing a minimum of 60% MeOH were analyzed by liquid chromatography–triple quadrupole tandem mass spectrometry (LC–MS/MS). Instrumentation consisted of an Agilent 1290 Infinity Binary Pump LC coupled to an Agilent 6495B triple quadrupole MS/MS with Jet Streaming Technology and electrospray ionization (ESI). Chromatographic separation was performed using an Agilent Poroshell 120 EC C18 column (2.1 × 100 mm, 1.9 μm). An Agilent Eclipse Plus C18 RRHD column (3.0 × 50 mm, 1.8 μm) was placed between the pump and the autosampler to remove any possible PFOS in the pump or mobile phases. Chromatographic separation was achieved using a gradient elution with a flow rate of 0.4 mL/min. Mobile phase A consisted of 10 mM ammonium acetate with 3% MeOH in MS grade water, and mobile phase B was 10 mM ammonium acetate with 20% acetonitrile in MS grade MeOH. The analytical column was held at a temperature of 50 °C during separation. Data acquisition was performed in dynamic multiple reaction monitoring mode using negative-mode ESI.

Agilent MassHunter Quantitative Analysis software Ver. B.10.0 was used for PFOS quantification. The software was used to calculate and plot peak area ratios of native analyte to labeled internal standard for quantitation. Using the calibration curve produced, the software calculated the PFOS concentration in each sample. Quality control samples included instrument blanks, instrument sensitivity checks at the limit of quantitation (LOQ), continued calibration verification check at the concentration of a midlevel calibrator, and a secondary source of PFOS. Quality control was performed with every set of samples and fell within the ±30% recovery requirement. The method limit of detection (LOD) and the LOQ for PFOS were 12 and 40 ng/L, respectively.

### PFOS measurement in zebrafish feed

Before the beginning of the exposures, the zebrafish feed (GEMMA Micro 75, 150, and 500) was analyzed for PFOS; all feed samples returned values less than the LOD of 75 ng PFOS/kg. The procedure for this analysis is provided in the Supporting Information, S1, Methods.

### External validation of measured PFOS exposure concentrations

Additional water samples were collected for 5% of the overall PFOS exposure water samples taken and sent to an independent PFAS-certified analytical testing facility (Test America, Denver, CO, USA) for analytical validation. The additional samples were collected at selected time points along with the standard 7-mL water samples taken for in-house analysis. For the external validation work, 250-mL aliquots of water were sent to Test America in opaque high-density polyethylene bottles. Samples were shipped on ice following a chain of custody protocol, with the temperature confirmed on arrival. Samples underwent solid-phase extraction at Test America using a modification of USEPA (2009) method 537.1. An additional subset of in-house analytical results was confirmed at a US Department of Defense (DOD) PFAS-accredited analytical laboratory (Eurofins, Lancaster, PA, USA) by evaluating sample splits in triplicate from three exposure concentrations (0.6, 3.2, and 20 μg/L) in a single validation test.

### Estimating endpoint-specific cumulative exposures

To provide the most accurate PFOS exposure values for each test endpoint across the survival, growth, reproduction, and VTG assessments, cumulative PFOS exposure was determined by averaging all measured PFOS values up to the time when a given endpoint was measured. As a means to eliminate biasing, the average cumulative exposure values, which had more sampling points in the first 6 weeks relative to the rest of the exposure (when samples were generally taken every 2 weeks), the cumulative PFOS exposures were calculated by first averaging any weekly collected samples to represent 2-week measurement values and then calculating averages of all the 2-week sample data up to the date at which each experimental endpoint was determined.

### Statistical analyses

Prior to conducting effects analyses for fish length, fish weight, reproduction, and VTG, each set of untransformed data was evaluated using Shapiro–Wilk analysis to test whether the data set was normally distributed (*p* = 0.05); if the data set was normally distributed, the Brown–Forsythe test was then used to test whether the data set had homogeneous variance (*p* = 0.05). Data sets meeting these assumptions were analyzed using parametric one-way ANOVA (*p* = 0.05) and Dunnett’s post hoc multiple comparisons tests to identify significant differences among controls and PFOS treatments (0.1, 0.6, 3.2, 20, and 100 μg/L). When data sets failed normality and/or homogeneity of variance tests, the effects of PFOS exposure were tested using nonparametric Kruskal–Wallis one-way ANOVA on ranks tests (*p* = 0.05) with Dunn’s post hoc multiple comparisons tests to establish pairwise differences among controls and PFOS treatments. All statistical analyses were conducted using SigmaPlot/SigmaStat Ver. 13.0 software (Systat). Given that parametric and nonparametric tests differ in statistical power, the type of analysis used for each dataset is summarized in the Supporting Information, S1, Table S3 to provide context for each test outcome. One caveat that should be considered when interpreting the individual one-way ANOVA tests for each experimental endpoint is the potential for increased false-positive identification propagated by chance due to performing multiple tests.

**FIGURE 2:**
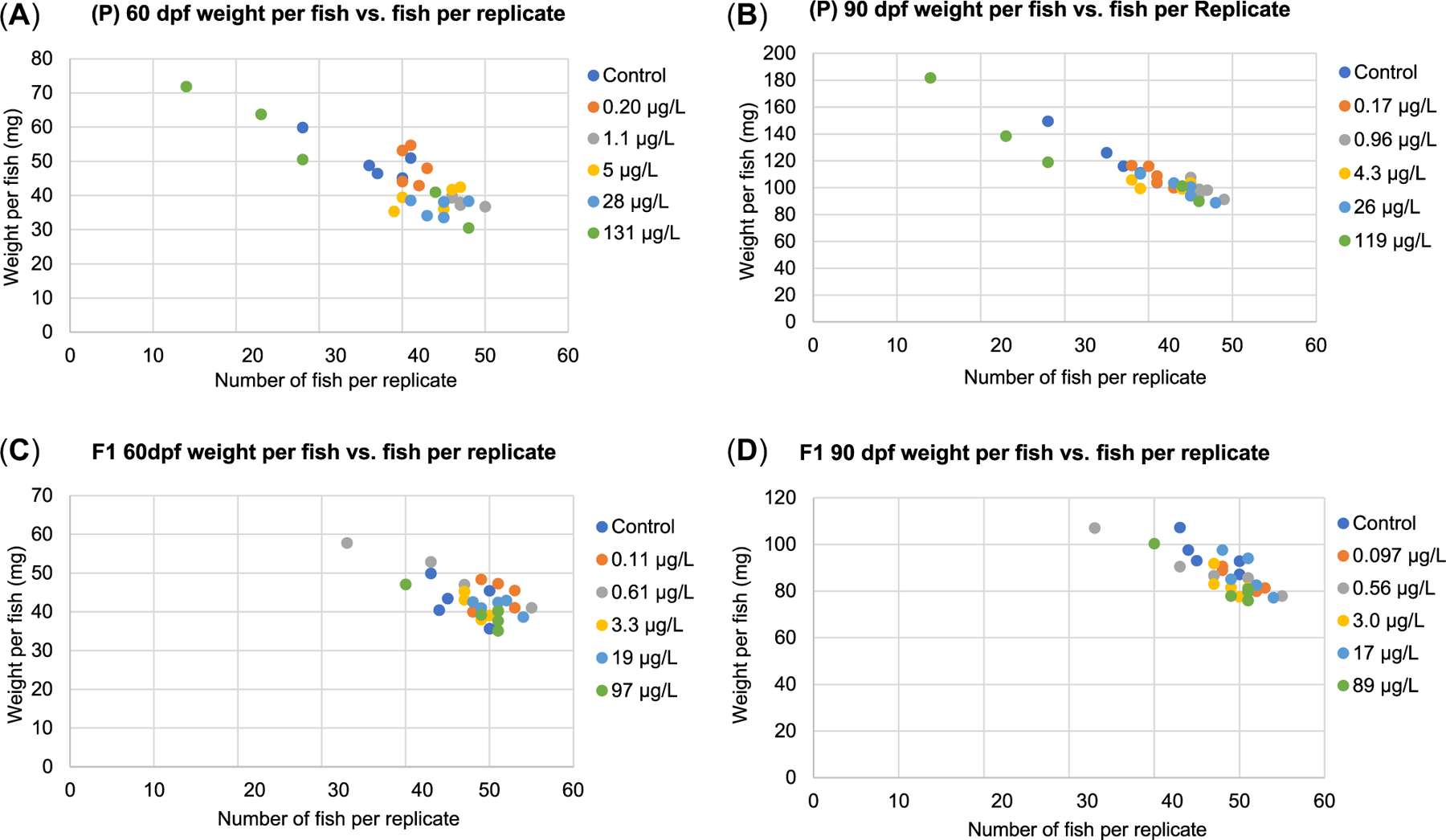
(A–D) Relationship between fish weight and number of fish present in each replicate at days 60 and 90 in the P and F1 generations. Analysis of covariance detected significant negative relationships (*p* < 0.05) between fish weight and fish number at each time point in each generation. Perfluorooctanesulfonic acid (PFOS) values represent cumulative measured concentrations in the exposure water reported as significant figures based on analytical method detection sensitivity. dpf = days post fertilization.

Because of observations that zebrafish growth can be highly sensitive to varied feeding regimes (Lawrence et al., 2012), an analysis of covariance (ANCOVA) was conducted to determine whether fish survival influenced growth observations in the present study. Significant negative relationships were observed among survival and fish weight endpoints (*p* = 0.05) when, under a uniform feeding regime, replicates containing fewer fish had greater individual fish growth within that replicate (Figure 2). Put simply, in chambers where survival was lower than 100%, the surviving fish received more food/fish than chambers in which no mortality occurred because a uniform amount of food was given to all chambers through the point at which the animals were regrouped at 111 dpf. Given these results, growth observations taken up to 90 dpf were tested on a per-replicate basis instead of per-individual, to evaluate growth accounting for the lower survival and uniform per-chamber feeding rates. This data analysis approach is consistent with the USEPA Whole Effluent Toxicity testing “biomass” approach for evaluating body weight in aquatic toxicity tests with replicates affected by <100% survival (Markle et al., 2000).

The effects of PFOS on fish whole-body wet weights were tested for the 60- and 90-dpf time points. Analyses were conducted using total fish weight/replicate, given the significant covariance observed between survival and growth, which effectively normalized the biomass generated to the standardized feeding ration applied across all treatments, regardless of survival. Given that Keiter et al. (2012) found differences in the growth responses by sex, fish weights at 180 dpf were analyzed individually for each sex with the data representing per-fish values because the fish were regrouped and provided uniform per-fish feeding rations after 111 dpf. The effect of PFOS on egg production/female and percentage of egg survival was investigated in the P and F1 generations. The egg production data represented the sum of all eggs produced/female across the eight individual breeding trials, and the egg survival data represented the average egg survival at 24 h across the eight breeding trials. Finally, the effects of PFOS on VTG concentration in male zebrafish whole-body tissues collected at 180 dpf in both the P and F1 generations were analyzed for significance using the nonparametric Kruskal–Wallis rank-sum test (*p* ≤ 0.05) in the native R stats package (R Core Team, 2023). Vitellogenin concentrations were calculated using the mean value/replicate (*n* = 5).

Effects of PFOS on zebrafish survival in the P and F1 generations were evaluated using survival data from 10, 15, 20, 30, 60, 90, 111, and 180 dpf. The PFOS effects on survival in the F2 generation were evaluated at days 10, 15, and 16 dpf. Because of extrabinomial variation (overdispersion), standard binomial distribution-based analyses of these survival data were not appropriate. The presence of overdispersion can be addressed by using a model with a fitted, greater-than-binomial variance, one option being the use of the beta-binomial distribution (Collett, 2003). This method was implemented with the R package “bayesnec” (Comprehensive R Archive Network, 2023) using a three-parameter log-logistic model (model = “ecxll3”) with family = beta_binomial2. The significance of effects was based on the slope parameter (beta) of the model being significantly greater than zero given its 95% credible limits.

### Statistical approach considerations

A variety of statistical approaches were initially identified and evaluated (e.g., simple one-way ANOVA ranging through complex multiway, repeated-measures ANOVA), and it was decided that individual analyses provided adequate statistical rigor and straightforward results interpretations. A caveat to this approach is that multiple tests can increase false-positives, which should be considered when significant effects are not prevalent or strong in magnitude. In general, the simple and more complex statistical models provided similar statistical inferences. For those that wish to analyze our results using alternative approaches, all data from the study are provided in the Supporting Information, S1, Materials. Both 10% lethal concentration (LC10) and 10% effect concentration calculations were executed for all endpoints (see the Supporting Information, S1, Methods for the approach); however, a lack of significant effects and an absence of clear concentration–response relationships resulted in nonsignificant model fits for the majority of the data and questionable reliability of the values that could be calculated.

## RESULTS

### Measured PFOS concentrations throughout the exposures

**FIGURE 3:**
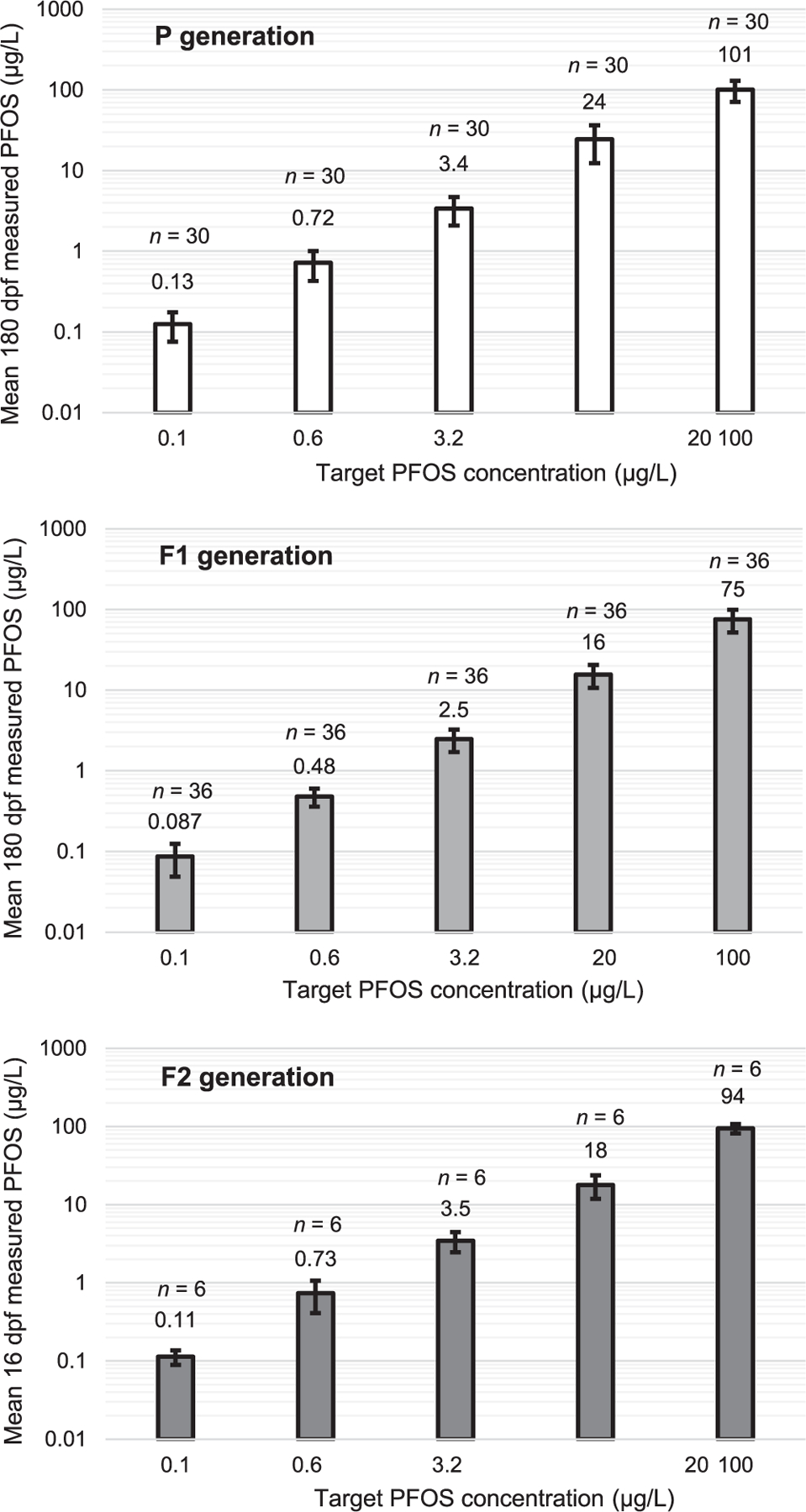
Bar charts represent the mean cumulative measured concentrations of perfluorooctanesulfonic acid (PFOS) in the exposure water for the P, F1, and F2 generation exposures at the completion of each experiment (180, 180, and 16 days post fertilization [dpf], respectively). Values above bars represent mean concentrations reported as significant figures based on analytical method detection sensitivity and error bars represent standard deviations. The *n* values represent the number of analytical sample replicates contributing to each PFOS measured concentration, including controls (not shown). All measurements of control samples returned values below the limit of detection, which was 12 ng/L.

For all exposures, the overall mean measured PFOS concentration was within ±30% of the nominal concentration (Figure 3). The variation in individual measured concentrations through time (Supporting Information, S1, Figure 5) indicated that 72% of measurements registered values within ±30% of the nominal concentration (see the Supporting Information, S1, Results for details). The cumulative measured PFOS exposure concentrations for all test endpoints are provided in Table 1, which serves as the reference point for attributing the best analytical estimation of PFOS exposure for each endpoint in the study.

### PFOS analytical chemistry validation

Comparison of the results between the in-house PFOS and independent PFOS analyses on sample splits indicated that differences in measured values were ≤26% (Supporting Information, S1, Table S4). The differences observed between in-house analytical results and those produced by the second DOD-certified PFAS testing facility (Eurofins) were 13, 0.32, and 8.8% for the PFOS nominal targets 0.6, 3.2, and 20 μg/L, respectively (Supporting Information, S1, Table S4).

### Zebrafish survival

**FIGURE 4:**
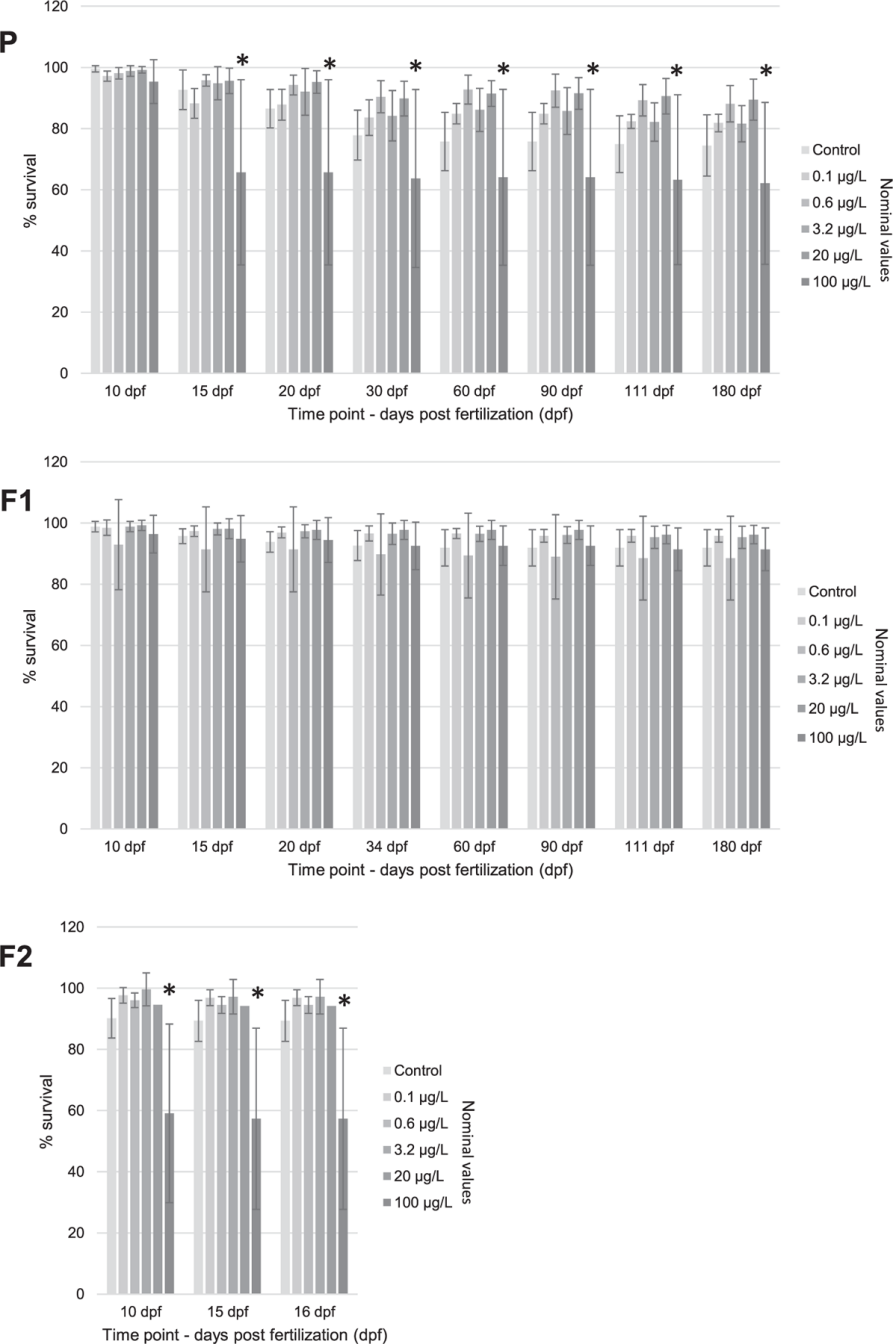
Zebrafish survival (%) in the P, F1, and F2 generations. Bars represent mean survival (*n* = 5), and error bars represent standard deviations. Exposure concentrations represent nominal values; the measured perfluorooctanesulfonic acid (PFOS) values are provided in Table 1 to provide analytically supported cumulative exposure concentrations. Asterisks represent statistically significant differences (*p* ≤ 0.01) relative to controls.

Survival at the highest PFOS exposure concentration (100 μg/L nominal) in the P generation was significantly reduced relative to all other treatments at 15 dpf and was also significantly reduced across all subsequent time points through 180 dpf (Figure 4). The measured PFOS concentration (205 μg/L) at 15 dpf was higher than nominal (100 μg/L), but concentrations more closely matched nominal targets after that time point (Table 1). Some mortality occurred in the P generation controls between 15 and 60 dpf, which stabilized to a final control survival of 75% (SD 10%) at 180 dpf, although survival was higher (82–90%) in the intermediate PFOS exposure treatments below 100 μg/L (nominal). By 180 dpf, survival in the highest PFOS exposure treatment (101 μg/L, measured) was 83% of the control and 73% relative to all lower PFOS exposures.

Zebrafish survival was consistently high in the F1 generation, regardless of PFOS exposure, with survival at 180 dpf of 92% (SD 7%) in the control, 91% (SD 7%) in the highest PFOS exposure, and 89 to 96% (SD 2–14%) in the other exposures. This lack of mortality for the highest exposure, in contrast to the P generation, may be associated with lower exposure concentrations, near nominal, early in the F1 generation exposure (Table 1).

Significantly increased mortality at the highest PFOS exposure concentration (94–97 μg/L, measured) was observed relative to all other treatments in the F2 generation, with reductions occurring at all time points tested and a 64% reduction relative to control survival observed at 16 dpf (Figure 4). Survival in the control at 16 dpf was 89% (SD 7%) and 96% (SD 4%) averaged over the intermediate PFOS exposure treatments below 100 μg/L (nominal). Relative to the P generation exposure, in which significant reductions in survival were associated with higher than expected PFOS concentrations in the early exposure time points, measured PFOS concentrations in the F2 generation exposure closely matched nominal PFOS targets (Table 1).

### Whole-body wet weights

**FIGURE 5:**
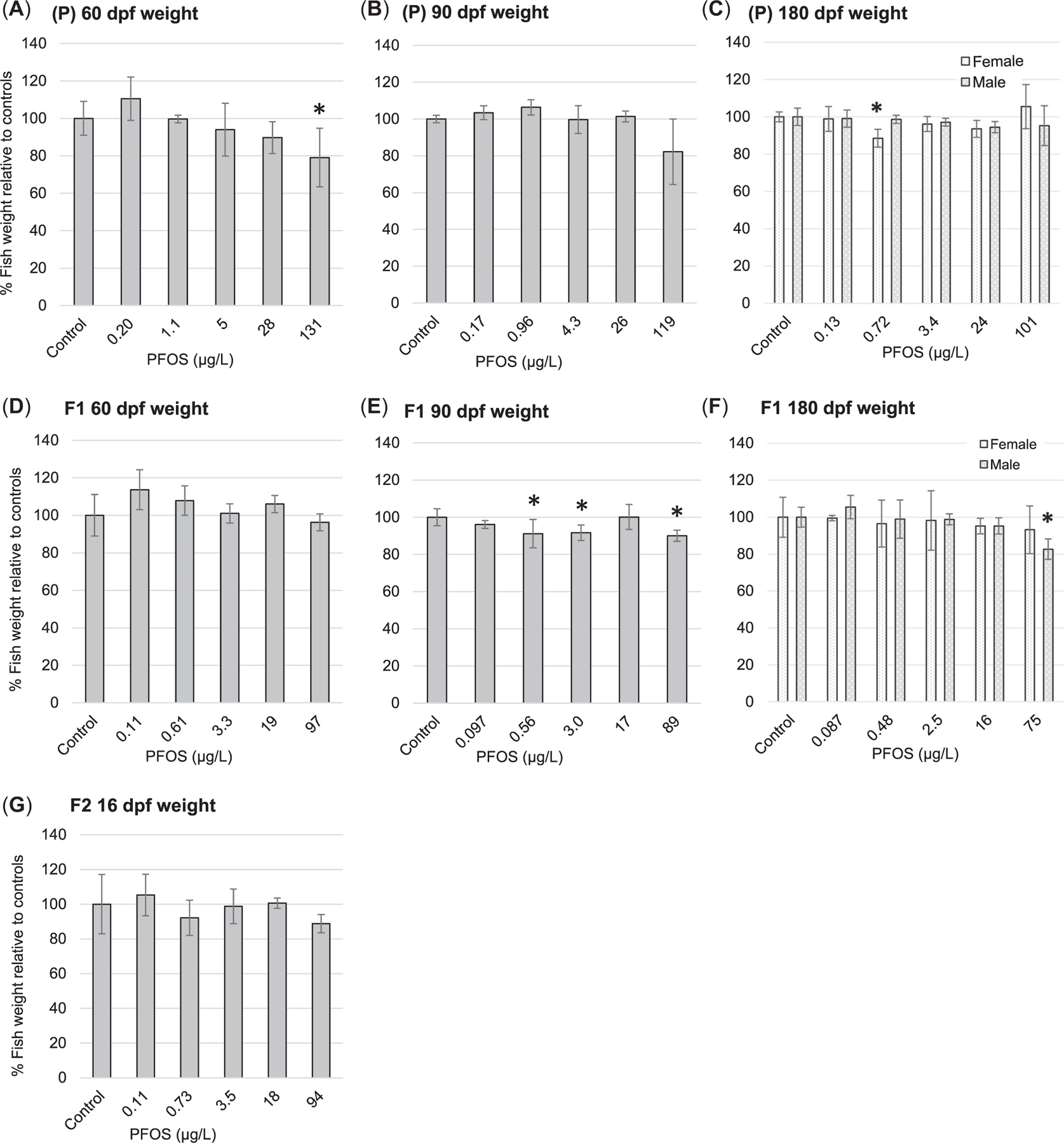
(A–G) Whole-body wet weights in the P, F1, and F2 generations representing the mean and standard deviation (*n* = 5) of the total fish weights in each replicate (relative to controls). Exposure concentrations represent measured cumulative mean perfluorooctanesulfonic acid (PFOS) concentrations through to the measurement endpoint reported as significant figures based on analytical method detection sensitivity. Asterisks represent statistically significant differences (*p* < 0.05) relative to controls. dpf = days post fertilization.

As described in the *Methods* section, ANCOVA identified a significant relationship between zebrafish survival and individual fish weights; replicates with decreased survival had fish that grew larger due to the consistent use of the single standardized per-chamber food ration (Figure 2). The zebrafish whole-body wet weight results presented for the 60- and 90-dpf time points in both the P and F1 generations (Figure 5) represent the sum of fish weights within each replicate to eliminate survival-dependent responses under a single food ration. In contrast, the 180-dpf values represent whole-body wet weights/fish because at 111 dpf, the number of fish were reduced in each replicate to achieve an even sex ratio consisting of 15 males and 15 females/tank. Starting at 111 dpf, feeding rations were applied based on food/surviving fish, thus eliminating differences in food availability among replicates.

In the P generation, the PFOS exposure caused a significant reduction in whole-body wet weight to 79% (SD 16%) of the control at the highest exposure concentration (131 μg/L, measured) at 60 dpf (Figure 5A). This degree of response remained at 90 dpf but was no longer significant relative to the control by 90 or 180 dpf (Figure 5B). At 180 dpf, the only significant response observed was a reduction in female body weights to 89% (SD 5%) of the control in the 0.72 μg/L (measured) exposure (Figure 5C). Mean body weights of the male and female fish exposed to the highest dose (101 μg/L, measured) at 180 dpf were 95% (SD 11%) and 106% (SD 12%) relative to controls.

The PFOS exposures in the F1 generation caused no significant effects on whole-body wet weights at 60 dpf (Figure 5D), whereas wet weights were significantly decreased to 91% (SD 8%), 92% (SD 4%), and 90% (SD 3%) of controls in the 0.56, 3.0 and 89 μg/L (measured) exposures, respectively, by 90 dpf (Figure 5E). By 180 dpf, the only significant effect on whole-body wet weight was a decrease in males to 83% (SD 6%) of controls at the highest PFOS exposure of 75 μg/L (measured; Figure 5F).

Finally, in the F2 generation, a decreased fish weight of 89% (SD 5%) relative to controls was observed at 16 dpf in the highest PFOS exposure concentration (94 μg/L, measured), but was not statistically significant (Figure 5G). It should be noted that two replicates that experienced high mortality in the 94 μg/L exposure (21% and 29% survival at 16 dpf) were removed from the F2 total fish weight analysis. If these two outliers were included as the lowest results (lowest ranked data) and evaluated nonparametrically, body weight in the 94 μg/L exposure was still not significantly different from controls.

### Reproduction

**FIGURE 6:**
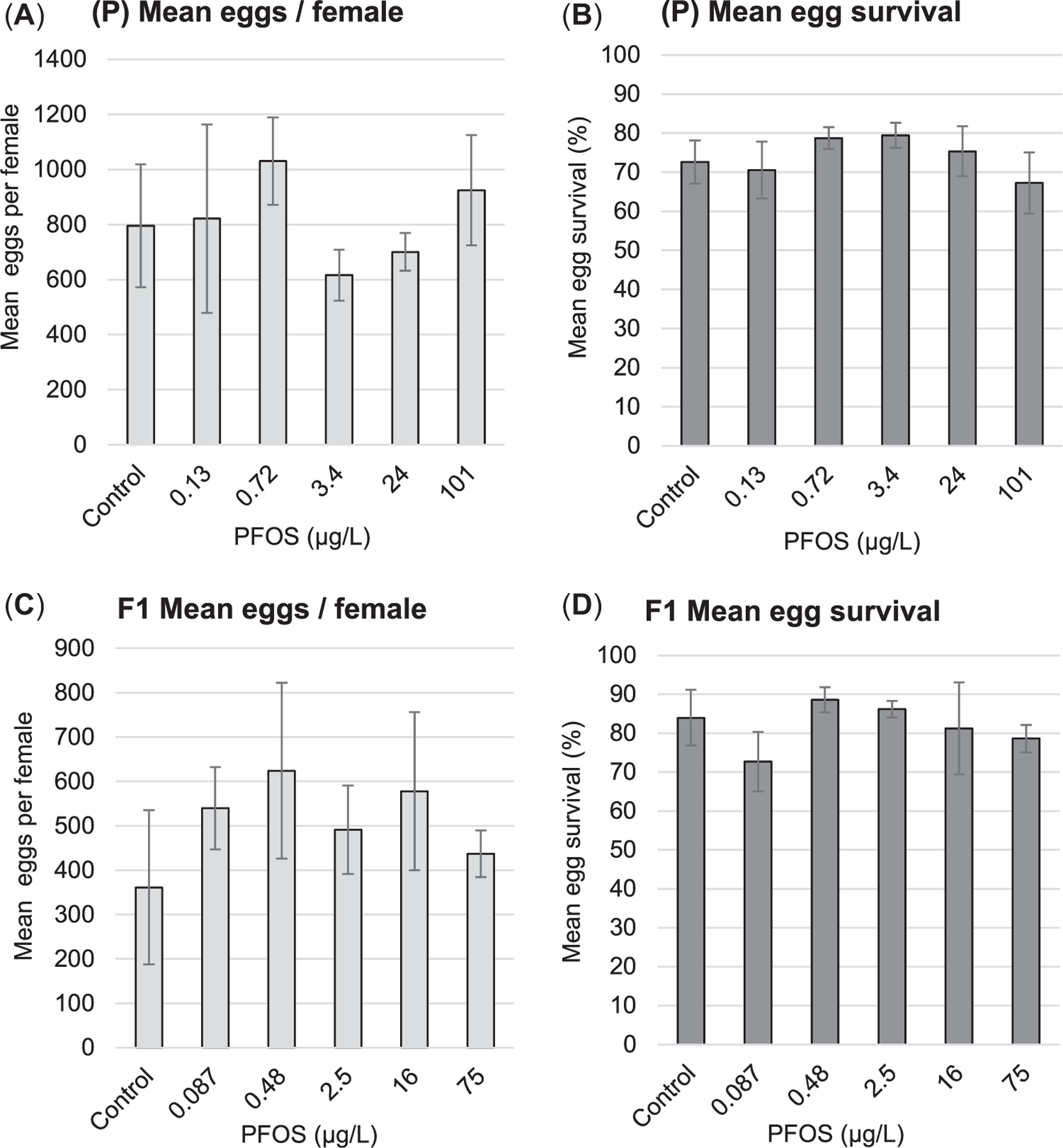
(A–D) Zebrafish egg production/female and egg survival (24 h) in the P and F1 generations, with bars representing means and error bars representing standard deviations (*n* = 5). The measurements represent the summation of 8 weekly breeding trials, and perfluorooctanesulfonic acid (PFOS) values are cumulative measured exposure concentrations reported as significant figures based on analytical method detection sensitivity. There were no statistically significant differences (*p* < 0.05) relative to controls.

The mean numbers of eggs produced/female and mean egg survival, each of which were summed across the eight breeding trials, were not significantly affected by the PFOS exposures in either the P or F1 generations (Figure 6). A trend of higher reproductive output was observed in the P generation compared with F1. Egg production has been observed to be variable in zebrafish (Castranova et al., 2011; Nasiadka & Clark, 2012); in the absence of any methodological differences between the P and F1 exposures, this may be the sole explanation for the difference in reproductive output among the generations in the present study. Regardless, egg production and egg survival did not differ from controls in any of the PFOS exposures.

### Male zebrafish whole-body VTG concentration

**FIGURE 7:**
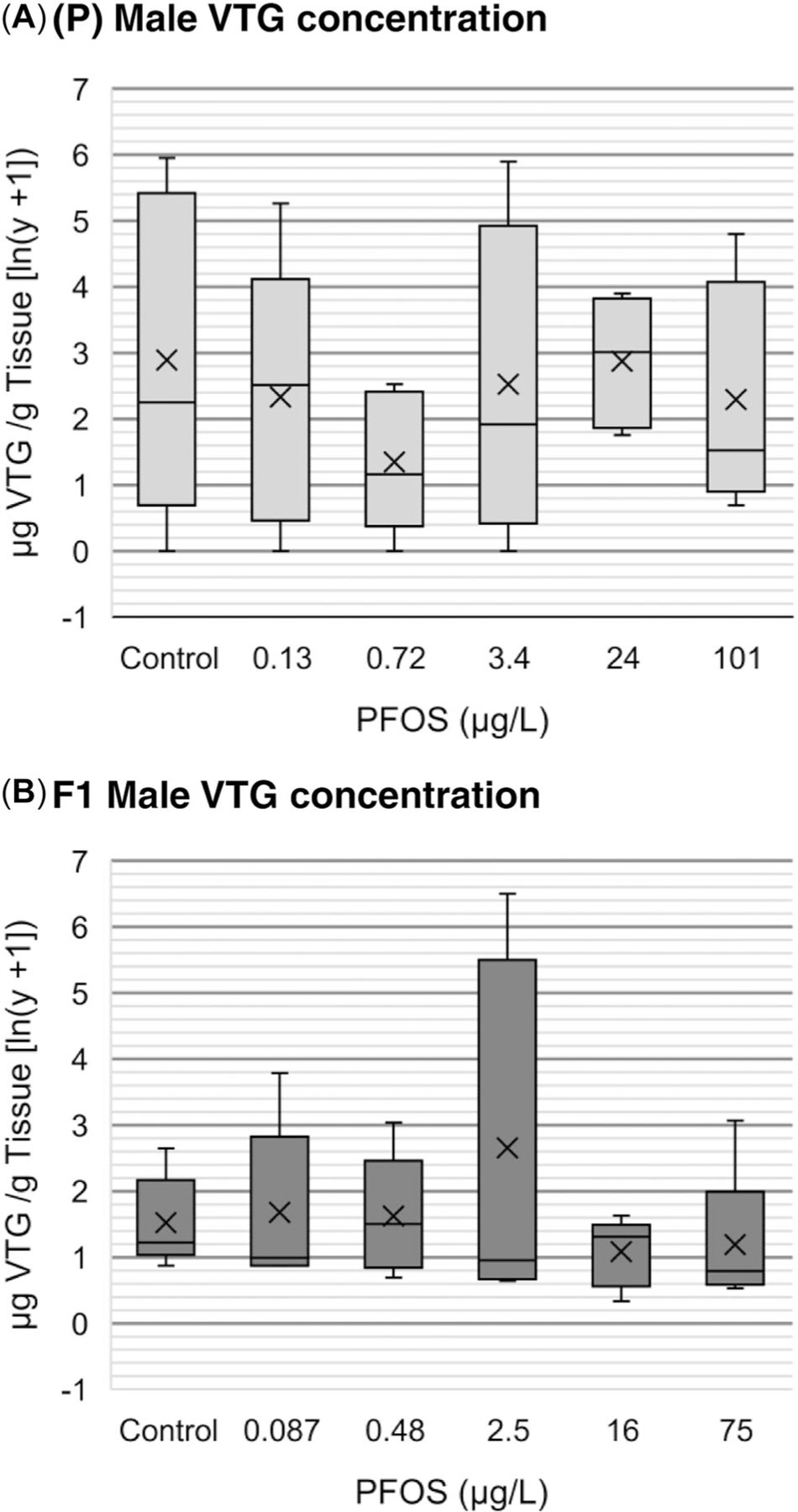
(A and B) Vitellogenin (VTG) concentrations in male zebrafish whole-body tissues collected at 180 days post fertilization (dpf) in both the P and F1 generations represented in a box and whisker chart in which *n* = 5. The “X” and the solid line within each bar represent the mean and median values, respectively. Perfluorooctanesulfonic acid (PFOS) values represent the cumulative measured water concentrations at 180 dpf reported as significant figures based on analytical method detection sensitivity. There were no statistically significant effects of PFOS relative to controls in either the P or F1 generations (*p* > 0.05).

The PFOS exposure had no significant effect on VTG concentrations relative to controls in male whole-body tissues measured at 180 dpf in either the P or F1 generations (Figure 7); the mean VTG concentration across all treatments was 39.5 μg/g (SE 14.2 μg/g, *n* = 60). For comparative purposes, the mean VTG concentration measured from whole-body tissues of control females, which produce VTG as part of normal reproductive physiology, was 70 420 μg/g (*n* = 4).

## DISCUSSION

### Zebrafish survival

In the present study, statistically significant adverse effects on zebrafish survival were observed in the P and F2 generations at the highest exposure concentrations of 205 and 96 μg/L, respectively, with most fish deaths occurring within 10 to 15 dpf (Figure 4). Similar mortality was not observed in the F1 generation, although this generation was exposed to 106.9 μg/L at early time points. Substantial variation in the effective PFOS concentrations causing significant lethal effects in zebrafish can be observed across previous studies. For example, in zebrafish embryo exposures lasting between 2 and 6 dpf, LC50 values for PFOS exposures ranged from 2200 to 68 000 μg/L (Huang et al., 2021; Krupa et al., 2022; Shi et al., 2008; Ulhaq et al., 2013; Vogs et al., 2019; Wasel et al., 2022; Zheng et al., 2012), the LOECs ranged from 3200 to 10 000 μg/L (Martinez et al., 2019; Ortiz-Villanueva et al., 2018; Truong et al., 2014), whereas the no-observed-effect concentrations (NOECs) ranged from 1530 to 20 000 μg/L (Huang et al., 2021; Mylroie et al., 2021). Survivorship responses to PFOS exposures extending beyond the embryonic life stage into subchronic and chronic exposures include reporting of a 30-dpf LC50 of 490 μg/L (Krupa et al., 2022), while Guo et al. (2019) and Bao et al. (2019) reported 21-dpf NOECs of 80 and 200 μg/L, respectively. Overall, the significant effects of PFOS exposures causing decreases in zebrafish survival at 205 μg/L and 15 dpf in the P generation and 96 μg/L at 10 dpf in the F2 generation observed in the present study represent the most sensitive subchronic survival response yet observed for zebrafish.

In studies extending beyond chronic exposures (>30 dpf) as well as multigenerational PFOS studies in zebrafish, significant mortality was not typically observed in exposures below 100 μg/L. For example, a recent study by Christou et al. (2021) exposed zebrafish during early development (0.25–5 dpf) to PFOS at 300 and 2060 μg/L (nominal), and no significant effects on survival were observed at 120 or 456 dpf. Similarly, Haimbaugh et al. (2022) administered PFOS at low concentrations of 0.024, 0.240, and 2.4 μg/L (nominal) to P-generation zebrafish in early developmental exposures (0.25–5 dpf) and identified no statistically significant effects in the P generation or subsequent unexposed generations (F1 and F2). Keiter et al. (2012) reported no significant zebrafish mortality in the P, F1, or F2 generations in 0.734 and 106.9 μg/L (measured) PFOS exposures. Although survival was not significantly impacted in the Keiter et al. (2012) study, survival was decreased by 10 to 40% relative to controls, which was similar in magnitude to the observations in the present study. Moreover, a higher PFOS exposure treatment at 267.6 μg/L (measured) caused P zebrafish to produce F1 offspring that displayed posthatch malformations ultimately resulting in 100% mortality (Keiter et al., 2012). Similarly, Wang et al. (2011) reported no significant mortality in P zebrafish exposed to PFOS at 5, 50, and 250 μg/L (nominal) for 5 months, but survival was 0% in F1 offspring produced by P fish exposed to the 250-μg/L PFOS treatment. The present study found the most sensitive lethal response to PFOS exposure in an extended-chronic exposure (>120 dpf) with a 180-dpf LOEC in the P generation of 101 μg/L. However, this lethal effect originated early in the exposure, manifesting at 15 dpf and persisting through 180 dpf. The next lowest LOEC observed for zebrafish survival in the literature for PFOS exposures of >120 dpf was 267.6 μg/L, observed by Keiter et al. (2012) in the F1 generation, while NOECs in the literature for exposures of this duration ranged from 75 to 2060 μg/L (Chen et al., 2013; Cheng et al., 2016; Christou et al., 2021; Cui et al., 2017; Wang et al., 2011).

### Zebrafish whole-body weight

Investigation of PFOS effects on zebrafish whole-body wet weights in the present study identified significant reductions relative to controls, with the most consistent effects occurring at the highest exposure treatment (75–131 μg/L, measured; Figure 5), with the highest magnitude effect being a reduction to 79% of the control weight. When considered in combination with significant body length reductions (Supporting Information, S1, Figure 6), males tended to be more sensitive to growth-related responses to PFOS than females. A few significant reductions in zebrafish whole-body wet weights were observed at intermediate PFOS exposure concentrations but these tended to be lower in magnitude, with body weights of ≥89% of controls; in addition, these effects did not follow monotonic dose–response relationships (Figure 5). The effects of PFOS on zebrafish body weights reported in the scientific literature vary considerably. For example, Guo et al. (2019) reported that PFOS exposures of 40 and 80 μg/L (nominal) caused significant body weight reductions in adult male zebrafish after 7, 14, and 21 days, reducing body weights to approximately 80% of controls. Krupa et al. (2022) noted a significant 30% reduction in zebrafish body weight at 260 μg/L (measured), but no significant effect at 92 μg/L (measured) in 30 dpf early life-stage exposures. Chen et al. (2016) observed exposure-duration–dependent significant differences in zebrafish body weights relative to controls in 250 μg/L (nominal) exposures, with a significant decrease observed at 21 dpf, a significant increase at 35 dpf, and finally, no effect by 42 dpf. Sex-specific differences in zebrafish body weight were reported by Du et al., (2008, 2009); exposure to 10, 50, and 250 μg PFOS/L (nominal) through 70 dpf caused no significant effects in females, but the highest exposure concentration caused significant decreases in males to 82% of control values. Similarly, Wang et al. (2011) observed sex-dependent differences in a 5-month continuous exposure; significant decreases in whole body wet weights were observed at 50 and 250 μg PFOS/L (nominal) in zebrafish males, but only at 250 μg/L in females.

In contrast to these studies, the multigenerational zebrafish study by Keiter et al. (2012), which administered PFOS exposures at 0.734, 106.9, and 267.6 μg/L (measured), reported significant reductions in whole-body wet weights for females in the P generation across all three PFOS treatments at 180 dpf, whereas no significant effects were found at any PFOS exposure at 30 and 90 dpf. In that study, the P-generation males had significantly reduced weights at the two highest exposure concentrations at both 90 and 180 dpf. Also, in that study, F1-generation zebrafish exposed to PFOS at 0.734 and 106.9 μg/L (measured) caused significant, but nonmonotonic body weight reductions, with significant effects at 0.734 μg/L but not at 106.9 μg/L at 30 dpf for both males and females. For both sexes, the weight reduction in the 0.734-μg/L exposure persisted at 90 dpf, but then did not differ significantly from controls by 180 dpf. In addition, significant body weight reductions were noted in the 106.9-μg/L exposure by 180 dpf in both sexes in the F1 generation (Keiter et al., 2012). In contrast to the Keiter et al. (2012) study, the present investigation did not find consistent effects of PFOS on body weight in <1-μg/L exposure concentrations. Also, the magnitude of body weight reduction in <1-μg/L PFOS exposures in the present study was at most 11% less than controls, whereas Keiter et al. (2012) reported multiple instances of body weight reductions by nearly 50% versus controls in the 0.734-μg/L exposure of the F1 generation.

### Reproduction

Investigation of zebrafish egg production and egg survival in the present study indicated no significant effect of PFOS in either the P or F1 generations (Figure 6). The multigenerational PFOS exposure conducted by Keiter et al. (2012) reported similar findings of no effects on egg production at 0.734 and 106.9 μg/L PFOS (measured) exposure concentrations. At the highest exposure concentration of 267.6 μg/L (measured) used by Keiter et al. (2012), there was no effect on P-generation egg production, but all F1 offspring died by 14 dpf. A similar response was observed in a study by Wang et al. (2011), in which P-generation zebrafish exposed to PFOS from 8 hpf through 5 months at 0, 5, 50, and 250 μg/L (nominal) exhibited no significant effects on egg production or hatching efficiency by 72 hpf. However, by 7 dpf there was a statistically significant mortality in the F1 generation from maternal fish exposed to 50 and 250 μg/L (nominal) PFOS (~40% and 100%, respectively), regardless of male exposure. A study by Chen et al. (2013) reported that P-generation zebrafish exposed to PFOS at 250 μg/L (nominal) from 21 to 120 dpf or from 1 to 120 dpf produced F1 offspring of which nearly 70 to 93% were malformed and died by 8 dpf. Finally, Bao et al. (2019) observed no significant effects on zebrafish egg production in adult females (120 dpf) exposed to PFOS at 2, 20, and 200 μg/L (nominal) for 21 days prior to reproduction assays. Overall, the results of both the present study and the supporting literature suggest that continuous multigenerational PFOS exposures ranging from 0.087 to 101 μg/L (measured) across P and F1 generations do not seem to affect zebrafish egg production or offspring short term (<7 dpf) survival through the F2 generation; however there is some uncertainty around the long-term (>7 dpf) survival of offspring maternally exposed to concentrations of PFOS >50 to 100 μg/L.

One mechanism by which PFOS could affect reproduction is by affecting signaling pathways controlled by the estrogen receptor (ER). A common in vivo indicator of a chemical’s potential to affect ER function in fish is induction of VTG, particularly in males, in which the lipoprotein typically is not detected and is not part of normal male reproductive physiology. Keiter et al. (2012) reported significant VTG increases in male zebrafish in a PFOS treatment of zebrafish at 0.734 μg/L and 90 dpf in both the P and F1 generations and at 180 dpf in the F1 generation, all of which displayed nonmonotonic dose–response relationships. Measurement of VTG in male zebrafish in the present study indicated that PFOS had no significant effects through 180 days of exposure in either the P or F1 generations (Figure 7). Our results are consistent with those of other studies in fish, indicating that PFOS is not an ER agonist, at least as indicated by induction of VTG (Ankley et al., 2005).

### Environmental context for PFOS exposures**/**effects

Based on the results of the present study, PFOS at 75 to 205 μg/L (measured) caused the most consistent adverse effects on zebrafish survival and growth endpoints in chronic, 180-dpf exposures. A comparison of our results with those testing sensitivity to PFOS in other freshwater aquatic species (as summarized in the USEPA [2022] Draft Aquatic Life Ambient Water Quality Criteria for PFOS) indicated that in chronic exposures, an effective concentration of approximately 100 μg/L falls near the middle of ranked freshwater genus mean chronic values, specifically 6th of 14 total genera evaluated. In terms of chronic PFOS exposures in freshwater fish species, the approximately 100 μg/L effective concentration was very similar to species mean chronic values (SMCVs) for Atlantic salmon (*Salmo salar*) and fathead minnow (*Pimephales promelas*), with respective values of >100 and 155.5 μg/L, but sixfold lower than swordtail fish (*Xiphophorus helleri*), with an SMCV of 599.7 μg/L.

Surface waters in the United States typically have PFOS concentrations orders of magnitude lower than the effective PFOS concentrations 75 to 205 μg/L (measured) observed in the present study. Specifically, a recent review by Jarvis et al. (2021) reported published PFOS concentrations measured in surface waters of the United States (encompassing both unimpacted and known impacted sites), with more than 90% of reported values falling below 0.2 μg/L and an overall range of 0.00016 to 8970 μg/L (median of 0.0055 μg/L). In a survey of surface water concentrations associated with US Air Force bases with a history of use of fire-fighting foams containing PFOS (a total of 256 sites across 85 installations), East et al. (2021) reported a mean concentration of 0.25 μg/L with 75th and 95th percentiles of 1.20 and 12.88 μg/L, respectively. Based on the results of that study and the observations from the present study, one might therefore anticipate a potential for effects in species with sensitivity similar to zebrafish at only a small fraction of sites (e.g., those most closely associated with known sources in waters impacted at the higher end of the exposure range).

## CONCLUSIONS

The present study was conceived with a rigorous design and implementation to provide robust and defensible freshwater aquatic effects characterization for multigenerational PFOS exposures in fish. The results indicate that significant lethal effects of PFOS exposure in zebrafish was typically expressed within 10 to 15 dpf at the highest PFOS exposure concentrations (100 μg/L, nominal; 94–205 μg/L, measured) with minimal additional mortality occurring thereafter through 180 dpf (Figure 4). Evidence of significant negative effects on zebrafish whole-body weights and lengths occurred most reliably at the highest PFOS exposure concentration (100 μg/L, nominal; 75–131 μg/L, measured), with no apparent compounding effects across generations (Figure 5 and Supporting Information, S1, Figure 6). Significant reductions in zebrafish weights were detected at lower PFOS concentrations, but these observations were infrequent, the dose–responses were typically nonmonotonic, and the magnitude of body weight loss was never more than 21% of the control. Finally, no significant effects of PFOS exposure were observed on egg production, egg survival, or reproductive endocrinology represented by whole-body VTG concentrations in males (Figures 6 and 7). Overall, the PFOS-induced increases observed in zebrafish mortality at approximately 100 μg/L (94–205 μg/L, measured) potentially represent the most impactful ecotoxicological response. Although PFOS exposure caused repeatable decreases in zebrafish whole-body weights at approximately 100 μg/L (75–131 μg/L, measured), the magnitude of body weight loss was never greater than 21% relative to the control (observed in the P generation at 60 dpf; Figure 5) and, ultimately, did not result in negative effects on egg production or egg survival (Figure 6). If mortality is selected as the basis for an aquatic effect benchmark, using the SMVC calculated based on USEPA (1985) guidelines, then the mean effective PFOS concentration for all significant lethal effects observed in the present study would be 117 μg/L (SE 8 μg/L, *n* = 9). If the aquatic effects benchmark were derived as the SMVC from all significant negative effects observed in the present study, regardless of nonmonotonic data structure and/or low likelihood of ecological impact, the final mean effective value would be 47 μg/L (SE 11 μg/L, *n* = 19).

## Supplementary Material

Supplement1

Supplement2

## Figures and Tables

**TABLE 1:** Cumulative measured perfluorooctanesulfonic acid (PFOS) exposure concentrations for all test endpoints including dates and exposure times as days post fertilization (dpf)

				PFOS target concentrations (μg/L) Measured ave. cumulative exposure (μg/L)				
Endpoint	Time point (dpf)	Cum. (no.)		0.1	0.6	3.2	20	100
Parental generation (P)								
Survival	10	0		Nom.	Nom.	Nom.	Nom.	Nom.
Survival	15	2	Ave	0.39	2.2	6.3	27	205
			SD	0.06	0.4	0.2	2	46
Survival	20	4	Ave	0.27	1.6	5.6	27	164
			SD	0.16	0.9	1.0	0	58
Survival	30	6	Ave	0.23	1.3	5.1	26	144
Growth	30		SD	0.14	0.8	1.1	2	54
Survival	60	12	Ave	0.20	1.1	5.0	28	131
Growth	60		SD	0.11	0.6	0.8	5	42
Survival	90	16	Ave	0.17	0.96	4.3	26	119
Growth	90		SD	0.10	0.58	1.3	6	40
Survival	111	18	Ave	0.16	0.92	4.2	30	121
			SD	0.10	0.55	1.3	11	37
Survival	180	30	Ave	0.13	0.72	3.4	24	101
Growth	180		SD	0.09	0.50	1.5	11	39
Reproduction	180							
Vitellogenin	180							
First filial generation (F1)								
Survival	10	0		Nom.	Nom.	Nom.	Nom.	Nom.
Survival	15	0		Nom.	Nom.	Nom.	Nom.	Nom.
Survival	20	2	Ave	0.11	0.64	2.9	20	107
			SD	0.01	0.07	0.4	1	24
Survival	34	4	Ave	0.12	0.67	3.3	21	102
Growth	34		SD	0.01	0.04	0.5	1	7
Survival	60	12	Ave	0.11	0.61	3.3	19	97
Growth	60		SD	0.01	0.08	0.3	3	6
Survival	90	16	Ave	0.097	0.56	3.0	17	89
Growth	90		SD	0.024	0.11	0.6	5	15
Survival	111	20	Ave	0.088	0.50	2.6	15	78
			SD	0.029	0.17	0.9	5	25
Survival	180	36	Ave	0.087	0.48	2.5	16	75
Growth	180		SD	0.028	0.12	0.7	5	20
Reproduction	180							
Vitellogenin	180							
Second filial generation (F2)								
Survival	10	2	Ave	0.11	0.59	3.0	15	96
			SD	0.03	0.14	1.2	10	19
Survival	15	4	Ave	0.11	0.61	3.2	17	97
			SD	0.02	0.10	1.0	8	14
Survival	16	6	Ave	0.11	0.73	3.5	18	94
Growth	16		SD	0.02	0.32	1.0	6	13

Each analytical measurement includes samples from at least two replicate exposure chambers for each PFOS treatment at each exposure time point (unless noted as “Nom.”, meaning that only nominal values are available due to lack of analytical values for a given time point). The measurement values reported in the table represent cumulative averages and standard deviations of measured values over time to represent the overall PFOS exposure in each generational exposure. The PFOS values are reported as significant figures based on analytical method detection sensitivity. Cumulative sample size (Cum. (no).) represents the total number of analytical samples contributing to each reported PFOS value when each PFOS exposure level included the Cum. (no.) number of samples, including controls. All measurements of control samples returned values below the limit of detection, which was 12 ng/L.

## Data Availability

Data for all experimental endpoints are provided in the Supporting Information S1, Materials in accompaniment to this open access paper.
