# Supplementary material for: Survival, Growth, and Reproduction Responses in a Three-Generation Exposure of the Zebrafish (Danio rerio) to Perfluorooctane Sulfonate
Source: Environ Toxicol Chem. Author manuscript; Available in PMC 2025 Jan 1. (PMC11131580; doi:10.1002/etc.5770)
Supplement: Supplement2 [file NIHMS1990584-supplement-Supplement2.docx]

**Supplemental Materials:**

**Title:** **Survival, Growth, and Reproduction Responses in a Three-Generation Exposure of the Zebrafish (*Danio rerio*) to Perfluorooctane Sulfonate (PFOS)**

**Authors:** Kurt A. Gust^1*^, J. Erik Mylroie^1^, Ashley N. Kimble^1^, Mitchell S. Wilbanks^1^, Catherine S. C. Steward ^2^, Kacy A. Chapman^3^, Kathleen M. Jensen^4^, Alan J. Kennedy^1^, Paige M. Krupa^1^, Scott A. Waisner^1^, Zacharias Pandelides^5^, Natalia Garcia-Reyero^1^, Russell J. Erickson^4^, Gerald T. Ankley^4^, Jason Conder^5^, David W. Moore^1^

**Affiliations:**

^1^US Army, Engineer Research and Development Center, Environmental Laboratory, Vicksburg, MS, USA

^2^Bennett Aerospace, Cary, NC, USA

^3^Oak Ridge Institute for Science and Education, Oak Ridge, TN, USA

^4^US Environmental Protection Agency, Great Lakes Toxicology and Ecology Division, Duluth, MN, USA

^5^Geosyntec Consultants, Costa Mesa, CA, USA

*Corresponding author: [kurt.a.gust@usace.army.mil](mailto:kurt.a.gust@usace.army.mil)

**METHODS:**

***Water Treatment System – Water Clean-Up Post-Exposure***

To capture PFOS from waste water generated during the experiment, exposure water exiting the test system first passed through a 1 µm particulate filter (Model #: H-F1001CF; Applied Membranes, Inc., Vista, CA) to remove any debris (fish food, feces, etc.), then through an activated-charcoal canister (CT-840; Enpress LLC, Eastlake, OH), and finally through three in-line canisters of Purofine PFA694 ionic resin (Supplemental Figure S1). The resulting treated water was collected in a 30,000L container for temporary storage. Prior to disposal, the treated water was analyzed for PFOS by a third-party certified analytical laboratory to confirm non-detection values for PFOS (method detection limit < 13 ng/L) in the treated water.

***Sample Size and Power Analyses***

To support the *a priori* sample size and power analyses for the zebrafish survival, growth, and reproduction ANOVA tests, published literature and in-house laboratory observations were used to estimate the variance associated with zebrafish survival, growth, and reproduction endpoints. The variance for the survival endpoint was determined based on several experiments executed in our laboratory (unpublished data). To establish the variance associated with the zebrafish growth endpoints, a study by Zhu et al. (2015) was utilized which included both male and female growth measurements for control fish as well as fish exposed to chemical stressors. For the reproductive assay, a multi-laboratory investigation of zebrafish reproductive parameters related to fish density (Castranova et al., 2011) was utilized where the mean of the observed variance within 8 laboratories was calculated using the fish stocking density that most closely matched our experimental apparatus. The variance for survival, growth, and reproduction were scaled to the effects level on a continuous scale from 0-100% where the measured variance for growth was 3.6%, the variance for reproduction was 11.7% and the variance for survival was 6.7% (Supplemental Table S1). Next, the desired sensitivity in effects detection (effect size) was established for each endpoint, where 15%, 30%, and 20% changes in growth, reproduction, and survival were targeted, respectively (Supplemental Table S1). The α for each test was set at p = 0.05 and the treatment number for the ANOVA test was set at 6 (control and 5 PFOS exposure concentrations). First, a sample size analysis was conducted for each of the endpoints where the minimum power was set at ≥ 0.8. The zebrafish reproductive test required the largest sample number to achieve test sensitivity and minimum power, requiring 5 replicates per treatment (Supplemental Table S1). Given that the survival, growth, and reproduction endpoints were to be collected from the same experimental test, an experimental design with a minimum of 5 replicates per treatment was required to fulfill the ANOVA statistical test requirements. Finally, power analyses were conducted for all experimental endpoints including 5 replicates per treatment (Supplemental Table S1) providing expected power for each test. All sample size and power analyses were conducted using SigmaPlot / SigmaStat v. 13.0 software (Systat Software, Inc. San Jose, CA)

***Preparation and Administration of Rotifers as Live Food for Zebrafish***

The rotifer stock and feed preparation method proceeded as follows: twenty million L-type rotifers were ordered from Reed Mariculture, Inc. (Pasadena, CA) and upon receipt the bags containing the rotifers in 30 parts per thousand water (ppt) were unpacked and allowed to rest on ice packs for ≈ 1 hour. After the resting period, a total of ≈10 million rotifers in water were combined into a 5 L plastic container and a small aliquot of the mixed rotifers was checked for viability and motility using a stereo microscope. Once viability was verified, the 10 million rotifers were collected 0.5 L at a time on a 53 µM sieve filter (Aquaculture Nursery Farms, Okeechobee, FL) and rinsed with pre-chilled (~8°C), 10 ppT Instant Ocean (IO) supplemented control water to remove dead algal feed. The rinsed rotifers were then washed off the sieve filter into a container containing 4 L of pre-chilled 10 ppt IO supplemented control water. The process was repeated with the mixed, unfiltered rotifers until all were rinsed and washed into the fresh pre-chilled 10 ppT control water. The volume in the “clean” rotifer stock was then increased to 4.5 L using cold 10 ppT control water and then the rotifers were fed 3 mL of RotiGrow® Plus rotifer feed (Reed Mariculture, Inc., Pasadena, CA). This process was repeated for the remaining ≈10 million unfiltered rotifers in two separate 5 L plastic containers and then both containers, each containing ≈10 million rotifers in 4.5 L of 10 ppT control water for a rotifer density of ≈2,200 rotifers/mL. Rotifers were stored at 8°C for a maximum of 1 week, and the containers were supplemented with ~1 mL RotiGrow® Plus every other day to help maintain maximum rotifer viability.

Aliquots of L-type rotifers were prepared as zebrafish food by collecting the needed volume of stock rotifers from one of the 5 L containers on a 53 µM sieve filter, rinsing with cold (8°C) 1 ppT IO supplemented control water, and then washing the rotifers into a beaker containing a volume of cold 1 ppT control water. The 1 ppT control water in the beaker was then increased to the necessary volume for the zebrafish meal and then allowed to warm at ambient temperature (26°C ± 0.5) for no less than 1 hour. Once the rotifer solution had warmed, the rotifer solution was aliquoted into 6 separate glass beakers (one for each of the exposure concentrations) at the volume needed to feed 5 zebrafish chambers with an extra 5-10 mL aliquoted to account for any dispensing error.

From 5 to 18 dpf, the zebrafish were fed 20 mL of rotifer suspension (containing ~40,000 rotifers) 2 x day (morning and evening), 10 mL of rotifers (containing ~20,000 rotifers) 1 x day (midday) and a small aliquot (9 mg) of GEMMA Micro 75, 2 x day (morning and evening) per replicate. From 18 – 30 dpf, the zebrafish were fed 20 mL rotifers 2 x day (morning and evening) and ~9 mg GEMMA Micro 75, 3 x day (morning, midday, and evening). The solutions were made at a salinity of 1.0 ppT to aide rotifer survival and motility. All chambers were cleaned prior to the daily water exchanges, which were conducted using a 50 mL serological pipette. Great care was taken to not disturb the zebrafish during the early stages of development to try to prevent mortality due to injury from the pipette. Starting at 15 dpf the water volume was gradually raised to a final volume of 2 L and the salinity was gradually reduced to 0.5 ppT.

***Zebrafish Exposure System Set-Up and Operation***

The flow-through exposure system was designed using a ZebTEC Stand-Alone Toxicology Rack (Tecniplast, Buguggiate, Italy) to house the fish (Supplemental Figure S2 and S3). The fish exposure system was calibrated manually to ensure equal delivery of water to all 30 chambers and then programmed to deliver water at a rate of 10.51 L/hr. This resulted in each chamber receiving ~120 mL of system water every 20 minutes with a 3-volume water exchanges every 24 hr. The PFOS treatment delivery system delivered control and PFOS treatments to the chambers using a total of six peristaltic pumps (Fisherbrand™ FH100M MultiChannel, Fisher Scientific, Pittsburg, PA, USA) which delivered ~12 mL of each PFOS treatment solution to each of five chambers on a single row on the exposure system (Supplemental Figure S2). A stratified random placement of fish chamber replicates was applied on a vertically-tiered, three-row exposure apparatus to place at least one chamber from each treatment on each row, but no more than two chambers per row (Supplemental Figure S3). PFOS solutions were transferred using PharMed® BPT Tubing (I.D. 0.056 in., O.D. 0.122 in., Cole-Parmer Instrument Co. Vernon Hills, IL, USA). A 50 mL polypropylene conical tube (Corning Life Science, Tewksbury, MA, USA) with a hole drilled above the chamber water line was used as a mixing device in each chamber to dilute the 10x PFOS stock with system water before the solution was delivered to each exposure chamber (Supplemental Figure S4). Volumes dispensed from the peristaltic pumps were checked at least 3 times per week.

A water temperature of 27^°^C ± 2 was maintained using heating elements in the exposure system sump. Automated addition of 30 g/L Instant Ocean or 30 g/L sodium bicarbonate to the sump maintained the target conductivity and pH ranges, respectively (Supplemental Table S2). Exposure chambers were individually aerated using one Whisper® Aquarium Pump 100 (Tetra, Blacksburg, VA, USA) for every 5 chambers. The salinity within the exposure system was slowly reduced from 0.5 ppT to 0.4 ppT ±0.03 and then maintained at that salinity for the rest of the 180 dpf exposures. The targeted exposure water parameters for the flow-through exposure were a pH 7.2 ± 0.5, conductivity of 750 – 850 µS/cm and a temperature of 27^°^C ± 2. Dissolved oxygen (target ≥ 60%) was monitored daily and total ammonia (target <1 ppm) was tested weekly. The mean conductivity for the static exposures, as explained in the feeding methods, was maintained higher than the during the flow-through exposure to improve the ability of the rotifers to stay in the water column, thus facilitating larval feeding.

***Production of First Filial (F1) and Second Filial (F2) Generations for Multigenerational PFOS Exposure***

At 179 dpf of the P and F1 exposures, the fish from each replicate were divided between two 1.7L Slope Breeding Tanks (Tecniplast, Buguggiate, Italy) where fish sex ratios approximating 1:1 were incorporated into each breeding chamber filled with control water the afternoon before the breeding event on 180 dpf. The males and females in each breeding chamber were separated by a plastic divider. The breeding chambers were placed on tables in a temperature-controlled environmental chamber (27°C ± 1), and the breeding chambers were individually aerated using one Whisper® Aquarium Pump 100 (Tetra, Blacksburg, VA, USA) for every 10 chambers. Fish were housed under these conditions overnight. The following morning, immediately after initiation of the diurnal light cycle, the dividers and airlines were removed from the chambers and the fish were allowed to spawn for ~45 minutes. Following the spawning period, the adult fish were removed and returned to their original replicate chamber on the exposure system. From each of the 2 breeding tanks per replicate, a batch of 250 embryos was collected, allowed to develop for ~1 hour and then surface sanitized using zebrafish embryo production methods described in the main manuscript text (Varga & Murray, 2016). Once sanitized, the two batches of embryos from each replicate were combined, screened for fertilization, and then at ~7 hpf, 3 batches of 25 fertilized embryos per replicate were transferred to 75 mL petri dishes containing 23 mL of the appropriate PFOS exposure treatment concentration in E2 media or control. These embryos were housed in static conditions at 28.5^o^C until 5 dpf. The slightly elevated temperature represents an in-house standard method to promote more rapid and robust development of the embryos. At 5 dpf, zebrafish from each replicate were transferred to a 3 L chamber containing 800 mL of the matching solution and exposure methods were carried out as described for the P generation except that the F2 generation was terminated at 16 dpf.

The back-up set of embryos produced for each the F1 and F2 generations were generated using the following methods. A minimum of 5 males and 5 females and a maximum of 8 males and 8 females from each replicate were retained in their original respective treatments / replicates on the fish exposure system beyond the 180 dpf exposure period and bred to produce embryos for both the F1 and F2 generations. At 184 dpf, all fish from each replicate were placed in individual static breeding chambers (as described previously) and breeding was initiated on day 185. Only one breeding chamber was used per replicate due to the reduced number of fish contributing to spawning; and therefore, one batch of 500 embryos was collected from each breeding chamber. From this point forward, the methods used for cleaning, screening, and exposure of embryos proceeded exactly as described for the primary embryo set described above.

***Completion of Each Generational Exposure***

At the completion of each generational exposure, the surviving fish were euthanized using an overdose of pH-adjusted MS-222 solution, growth measurements were recorded (see Effect of PFOS on Growth section), and either the whole bodies or specific tissues were harvested and archived for investigations of PFOS bioaccumulation, histopathology, and transcriptomics investigations to be described in future manuscripts. The takedown of each generation was conducted by harvesting the fish from each replicate following the randomly assigned replicate number.

***Zebrafish Body Length Measurements***

At 30, 60, and 90 dpf in the P generation and at 34, 60, and 90 dpf in the F1 generation, the lengths of every fish in each replicate were measured by collecting subgroups of 8 to 12 zebrafish and placing them in petri dishes containing ~ 20 mL of treatment water and recording video for 30 seconds. Fish length was measured by analyzing still images from videos collected using the DanioVision (Noldus, Wageningen, Netherlands) observation chamber. DanioScope software (v. 1.0.109, Noldus) was used to capture still images from each video from which the total body length of each fish was measured. An EVOS™ calibration slide (Applied Image Inc., Rochester, NY, USA) was used as a reference to provide a µM length scale for a calibration profile. Fish sex was not determined for the 30, 60 and 90 dpf measurements. The 180 dpf length measurements for both the P and F1 generations proceeded using the euthanized fish where fish sex was recorded and then the body length measured using a dissecting ruler. At the termination of the F2 generation (16 dpf), lengths for all surviving fish per replicate were captured for live fish using the DanioVision method. Similar to the whole-body weight analyses, effects of PFOS on zebrafish length were conducted on composite length measurements representing the sum of fish lengths for each replicate given the significant co-variance observed among survival and growth. Also, similar to the weight measurements, fish lengths at 180 dpf were analyzed individually for each sex.

***Single Timepoint Water Quality Parameters***

Two water quality parameters, total alkalinity and total hardness, were measured at one timepoint at the beginning of the P generation to establish background levels. Both total alkalinity and total hardness were measured with the SMART® 3 Colorimeter (Product #: 1910; LaMotte Company, Chestertown, MD) using the accompanying kits for total alkalinity and hardness (Total Alkalinity – UDV; Product #: 4318-J; Total Hardness – UDV; Product #: 4309-J; LaMotte Company, Chestertown, MD).

***PFOS Measurement in Zebrafish Feed***

The zebrafish feed extraction method was a modified method of ASTM D7968-17a for soils (ASTM, 2017). 2 g of feed was extracted by adding 10 mL of 50:50 (v/v) H_2_O:MeOH adjusting pH to ~9, and tumbling for 1 h, before centrifuging at 2800 rpm for 10 min and collecting the supernatant by passing it through a 0.2 micron nylon syringe filter (Cytiva Whatman) that was pre-rinsed with two 5 mL aliquots of acetonitrile and one 5 mL aliquot of MeOH. The final extract was adjusted to a pH of ~3. An aliquot was removed for analysis and internal standard was added before quantitation.

***LC10 and EC10 Calculations (Results not reported due to lack of model fit)***

Lethal concentrations inducing a 10% population response (LC10) and effective concentrations inducing a 10% sublethal population response (EC10) values were calculated for experimental data by first setting control values to unity to allow a proportional general linear model (GLM) curve fit to be executed using R Software V 4.0.3 (RStudio, Boston, MA, USA). Most datasets did not produce discernable concentration-response relationships due to insufficient reductions in measurement endpoints at the higher exposure concentrations. Therefore, LC10 or EC10 values could not be generated for the majority of the experimental datasets.

**RESULTS:**

***Analytical Chemistry***

Measurement of PFOS concentrations for individual sampling periods indicated that 60%, 70%, and 85% of zebrafish exposures in the P, F1, and F2 generations, respectively, fell between ±30% of target concentration (Supplemental Figure S5). Further, the individual PFOS sample measurements for water samples taken from each treatment, 0.1, 0.6, 3.2, 20, and 100 µg/L, fell within ±30% of targeted concentration for 73%, 74%, 63%, 70%, and 77% of samples, respectively.

***Zebrafish Body Length***

The covariance observed between zebrafish survival and fish weights (Figure 2) was also manifested in the body length measurements; thus, sum body lengths per replicate were analyzed for the 30, 60, and 90 dpf sampling time points for the P and F1 generations and at 16 dpf in the F2 generation (Supplemental Figure S6). The 180 dpf body length measurements represent per-individual mean values given the reduction in number of zebrafish to 15 males and 15 females per replicate exposure at 111 dpf when the feeding ration was normalized to the number of surviving fish.

In the P generation, the PFOS exposure caused decreased total fish lengths to a minimum of 83% (S.D. 32%) of control lengths at the highest PFOS exposure concentration at 30, 60, and 90 dpf, however the effect was not significant (Supplemental Figure S6A-S6C). The only significant PFOS effects observed across these time points were increases in total fish lengths at 60 and 90 dpf where lengths were 123% (S.D. 3%) and 124% (S.D. 4%) of controls at 1.2 µg/L and 1.0 µg/L (measured), respectively. Significant, yet relatively minor reductions in total body lengths were observed in male zebrafish at 180 dpf where body lengths were 97% (S.D. 1%) and 96% (S.D. 2%) of controls in the highest two PFOS exposure concentrations of 24.5 µg/L and 101.0 µg/L (measured), respectively (Supplemental Figure S6D).

The PFOS exposures in the F1 generation caused no statistically significant effects and no discernable trends in total body lengths through the 34, 60, and 90 dpf sampling time points (Supplemental Figure S6E- S6G). The only statistically significant effect of PFOS observed in the F1 generation occurred at the highest exposure concentration (75 µg/L, measured) where male zebrafish lengths were reduced to 93% (S.D. 2%) of the control (Supplemental Figure S6H).

Finally, in the F2 generation, a statistically significant decrease in total fish lengths was observed at 16 dpf where the highest PFOS exposure concentration (94 µg/L, measured) caused fish lengths to be reduced to 83% (S.D. 1%) of the control (Supplemental Figure S6I). It should be noted that two replicates which experienced high mortality in the 94.4 µg/L exposure (21% and 29% survival at 16 dpf) were removed from the F2 total fish length analysis.

**DISCUSSION:**

***Zebrafish Body Length***

Investigation of zebrafish body lengths in the present study indicated no significant negative effects of the PFOS in the P or F1 generation from 30-90 dpf, but there was a decrease in males at the highest two exposure concentrations at 180 d in the P generation and at the highest exposure concentration in the F1 where lengths were reduced to a minimum of 93% of the control (Supplemental Figure S6). The greatest magnitude of body length reduction was observed in the F2 generation exposed to 100 µg/L PFOS by 16 dpf where lengths were 83% of the control. Previous studies also have examined the effects of PFOS in zebrafish embryo / early larval exposures (ranging from 96-132 hpf). For example, Dang et al. (2018) reported reductions in 50, 500 or 5,000 μg PFOS/L (nominal) exposures; Hagenaars et al. (2011) reported significant reductions at 1, 5, and 10 mg/L but not 0.1 and 0.5 mg/L (nominal); Shi et al. (2008) reported significant reductions at 3 and 5 mg/L but not at 0.1, 0.5, and 1 mg/L (nominal); and Christou et al. (2021) reported no significant effects of PFOS at 275 and 1,915 μg/L (nominal). Jantzen et al. (2016) showed that zebrafish had significantly decreased body lengths in response to PFOS at 100 and 1000 μg/L (nominal) exposures at 5 dpf but no significant effects by 7 and 14 dpf timepoints, indicating that PFOS effects on zebrafish body length may be developmental-stage dependent. As was observed for zebrafish whole-body weights, sensitivity of zebrafish to PFOS exposure on body lengths varied considerably in the scientific literature. For example, Guo et al. (2019) observed significantly decreased body lengths in adult male zebrafish exposed to 80 μg/L (nominal) PFOS exposure for 14 and 21 days, though length reductions were somewhat minor (approximately 10-15% reduced relative to controls). In contrast, a zebrafish exposure conducted by (Shi et al., 2009) to PFOS at 100, 200 and 400 μg/L (nominal) reported significantly decreased body lengths only the highest exposure concentration at 15 dpf with length reductions of approximately 20% of the control in that treatment. Further, Chen et al. 2016 observed time-point-specific significant differences in zebrafish body length relative to controls in 250 μg/L (nominal) exposures where body lengths were decreased, increased, or unchanged at 21, 35, and 42 dpf, respectively. Sex-dependent differences in PFOS sensitivity were also observed where, in a 70d zebrafish exposure to PFOS at 10, 50 and 250 μg/L (nominal), the highest exposure concentration caused significant decreases in male length, but not in the females (Du et al., 2008 and 2009).

Finally, in the study closest to our design, Keiter et al. (2012) also reported several instances of PFOS-induced reductions in zebrafish body length. In that study, significant body length reductions were found in the P generation for PFOS exposures at 0.734, 106.9, and 267.6 µg/L (measured) in females at 180 dpf, but no significant effects for any concentration of PFOS on female length were measured at 30 or 90 dpf. In males there was no statistically significant effect of exposure to PFOS on length at 30 dpf, at 90 dpf there was a statistically significant decrease in length of 5-8% for all concentrations tested, and a statistically significant decrease in length of fish exposed to 106.9 and 267.6 µg/L (measured) for males (10-13%) by 180 dpf. In contrast to those results, the present study only identified statistically significant reductions in P generation body lengths in males at 24.5 and 101 µg/L (measured) at 180 dpf (Supplemental Figure S6) where reductions were relatively minor with a minimum body length within 96% of the control. In the F1 generation, Keiter et al. (2012) reported multiple instances of significant body length reductions in male and female zebrafish body lengths in response to PFAS at 0.734 µg/L (measured) across multiple exposure timepoints. The present study observed significant reductions in F1 body lengths only in males at the highest PFOS exposure level of 75 µg/L (measured) and again only at the highest exposure concentration of 94 µg/L (measured) in the F2 generation (Supplemental Figure S6).

**LITERATURE CITED**

ASTM. (2017). *Standard Test Method for Determination of Polyfluorinated Compounds in Soil by Liquid Chromatography Tandem Mass Spectrometry* *(LC-MS/MS)* (ASTM Standard D7968-17a). ASTM International, West Conshohocken, PA.

Castranova, D., Lawton, A., Lawrence, C., Baumann, D., Best, J., Coscolla, J., Doherty, A., Ramos, J., Hakkesteeg, J., Wang, C., Wilson, C., Malley, J. M., & Weinstein, B. (2011). The effect of stocking densities on reproductive performance in laboratory zebrafish (*Danio rerio*). *Zebrafish,* 8(3), 141-6. DOI: 10.1089/zeb.2011.0688.

Chen, J., Wang, X., Ge, X., Wang, D., Wang, T., Zhang, L., Tanguay, R. L., Simonich, M., Huang, C., & Dong, Q. (2016). Chronic perfluorooctanesulphonic acid (PFOS) exposure produces estrogenic effects in zebrafish. *Environmental Pollution,* 218, 702-708.

Christou, M., Ropstad, E., Brown, S., Kamstra, J. H., & Fraser, T. W. K. (2021). Developmental exposure to a POPs mixture or PFOS increased body weight and reduced swimming ability but had no effect on reproduction or behavior in zebrafish adults. *Aquat. Toxicol.,* 237, 105882.

Dang, Y., Wang, F., & Liu, C. (2018). Real-time PCR array to study the effects of chemicals on the growth hormone/insulin-like growth factors (GH/IGFs) axis of zebrafish embryos/larvae. *Chemosphere*, 207, 365-376.

Du, Y., Shi, X., Liu, C., Yu, K., & Zhou, B. (2009). Chronic effects of water-borne PFOS exposure on growth, survival and hepatotoxicity in zebrafish: A partial life-cycle test. *Chemosphere,* 74, 723-729.

Du, Y., Shi, X., Yu, K., Liu, C., & Zhou, B. (2008). Chronic effects of waterborne PFOS exposure on growth, development, reproduction and hepatotoxicity in zebrafish. In Y. Murakami, K. Nakayama, S.-I. Kitamura, H. Iwata & S. Tanabe (Eds.), *Interdisciplinary Studies on Environmental Chemistry-Biological Responses to Chemical Pollutants* (pp. 37–54). TERRAPUB.

Guo, J., Wu, P., Cao, J., Luo, Y., Chen, J., Wang, G., Guo, W., Wang, T., & He, X. (2019). The PFOS disturbed immunomodulatory functions via nuclear Factor-κB signaling in liver of zebrafish (*Danio rerio*). *Fish Shellfish Immunol.,* 91, 87-98.

Hagenaars, A., Vergauwen, L., De Coen, W., & Knapen, D. (2011). Structure-activity relationship assessment of four perfluorinated chemicals using a prolonged zebrafish early life stage test. *Chemosphere*, 82, 764-772.

Jantzen, C. E., Annunziato, K. A., Bugel, S. M., & Cooper, K. R. (2016). PFOS, PFNA, and PFOA sub-lethal exposure to embryonic zebrafish have different toxicity profiles in terms of morphometrics, behavior and gene expression. *Aquat. Toxicol.*, 175, 160-170.

Keiter, S., Baumann, L., Farber, H., Holbech, H., Skutlarek, D., Engwall, M., & Braunbeck, T., (2012). Long-term effects of a binary mixture of perfluorooctane sulfonate (PFOS) and bisphenol A (BPA) in zebrafish (*Danio rerio*). *Aquat. Toxicol*., 118-119, 116-129.

Shi, X., Du, Y., Lam, P. K., Wu, R. S., & Zhou, B. (2008). Developmental toxicity and alteration of gene expression in zebrafish embryos exposed to PFOS. *Toxicol. Appl. Pharmacol.,* 230, 23-32.

Shi, X., Liu, C., Wu, G., & Zhou, B. (2009). Waterborne exposure to PFOS causes disruption of the hypothalamus-pituitary-thyroid axis in zebrafish larvae. *Chemosphere*, 77, 1010-1018.

Varga, Z. M., & Murray, K. N. (2016). Health monitoring and disease prevention at the Zebrafish International Resource Center. In W. H. Detrich 3rd, M. Westerfield, & Z. I. Leonard (Eds.), *The Zebrafish: Genetics, Genomics, and Transcriptomics* (Vol 135: Methods in Cell Biology, 4th ed., pp. 535-551). Academic Press.

Zhu, Y., Ma, X., Su, G., Yu, L., Letcher, R. J., Hou, J., Yu, H., Giesy, J. P., & Liu, C. (2015). Environmentally relevant concentrations of the flame retardant tris(1,3-dichloro-2-propyl) phosphate inhibit growth of female zebrafish and decrease fecundity. *Environ. Sci. Technol.,* 49, 14579-14587.

Supplemental Table S1. *A priori* sample size and power analyses for prospective parametric ANOVAs including 6 treatment levels (control and 5 PFOS exposure concentrations) for survival, growth, and reproduction endpoints in zebrafish. The sample size analysis was used to identify the minimum number of replicates needed to achieve a minimum power of 0.8 for all endpoints. The sample-size analysis indicated that the reproduction end point required the greatest number of replicates (n = 5) to achieve the desired test sensitivity paired with the minimum required statistical power. Given that all endpoints would be collected from the same experiment, separate power analyses were conducted for all endpoints using the sample size of 5 to calculate statistical power.

 Supplemental Table S2. The cumulative water quality measurements for the P, F1, and F2 generations. Water quality measurements show the mean, maximum, and minimum measurements from days 6 – 180.

^1^Conductivity values during the static exposure were higher than those for the flow-through exposures to improve the ability for rotifers to stay in the water column and facilitate zebrafish larval feeding.

^2^Temperature did not vary among treatments during exposures.

^3^Total Ammonia readings were not recorded during the F2 static exposure.

^4^Measurements for alkalinity and hardness taken at a single timepoint at the start of the P generation exposure for background values. Measurements are a mean of values taken from two random replicates from each of the treatment groups.

Supplemental Table S3. Summary of statistical methods and results for all statistical analyses conducted in the present study. All pairwise tests represent comparisons relative to controls where green shading represents an increased relative to controls and red shading represents a decreased response relative to controls.

Supplemental Table S4. Third party validation of PFOS analytical chemistry analyses. October samples were from the P exposure and the other two from F1 exposure.

| **Nominal Conc. (µg/L)** | **ERDC Analytical Results** | | | | | **Test America Analytical Results** | | | | | **Percent**  **Difference** |
| --- | --- | --- | --- | --- | --- | --- | --- | --- | --- | --- | --- |
|  | **Oct-20**  **(µg/L)** | **May-21**  **(µg/L)** | **Jul-21**  **(µg/L)** | **Average** | **RSD**  **(%)** | **Oct-20**  **(µg/L)** | **May-21**  **(µg/L)** | **Jul-21**  **(µg/L)** | **Average** | **RSD**  **(%)** |  |
| 0.1 | 0.086 | 0.036 | 0.073 | 0.065 | 33 | 0.050 | 0.042 | 0.054 | 0.049 | 10 | 26 |
| 0.6 | 0.54 | 0.38 | 0.50 | 0.47 | 14 | 0.53 | 0.32 | 0.46 | 0.44 | 20 | 6.6 |
| 3.2 | 1.9 | 1.9 | 2.9 | 2.2 | 21 | 2.0 | 1.7 | 2.8 | 2.2 | 21 | 3.0 |
| 20 | 19 | 12 | 17 | 16 | 18 | 18 | 11 | 17 | 15 | 20 | 13 |
| 100 | 69 | 45 | 117 | 77 | 39 | 86 | 38 | 130 | 85 | 44 | 9.9 |

| **Nominal**  **Conc.**  **(µg/L)** | **ERDC Analytical Results** | | | | | **Eurofins Analytical Results** | | | | | **Percent**  **Difference** |
| --- | --- | --- | --- | --- | --- | --- | --- | --- | --- | --- | --- |
|  | **1 (µg/L)** | **2 (µg/L)** | **3 (µg/L)** | **Average** | **RSD (%)** | **1 (µg/L)** | **2 (µg/L)** | **3 (µg/L)** | **Average** | **RSD (%)** |  |
| **0.6** | 0.66 | 0.65 | 0.61 | 0.64 | 3.4 | 0.49 | 0.62 | 0.58 | 0.56 | 9.7 | 13 |
| **3.2** | 3.3 | 2.9 | 3.1 | 3.1 | 5.3 | 2.4 | 3.6 | 3.3 | 3.1 | 16 | 0.32 |
| **20** | 17 | 20 | 16 | 18 | 9.6 | 14 | 17 | 18 | 16 | 10 | 8.8 |

**B.**

**C.**

**A.**

Supplemental Figure S1. Overview of PFAS water treatment system for treating RO water with Purofine® PFA694 (Purolite LLC, King of Prussia, PA) ionic resin to remove any residual contaminants (including fluorinated chemicals) from the water prior to entry to the exposure system (Panel A). The treatment train for experimental exposure water exiting the exposure system included passage through a 1 µm particulate filter (Model #: H-F1001CF; Applied Membranes, Inc., Vista, CA), an activated-charcoal canister (CT-840; Enpress LLC, Eastlake, OH), and finally through three in-line canisters of the Purofine PFA694 ionic resin (Panel B). The resulting treated water was collected in a 30,000L container for temporary storage. Prior to disposal, the treated water was analyzed for PFOS by a third-party certified analytical laboratory to confirm non-detection values for PFOS (method detection limit < 13 ng/L) in the treated water (Panel C).

Supplemental Figure S2. Diagram of exposure system used for multi-generational PFOS exposures in zebrafish.


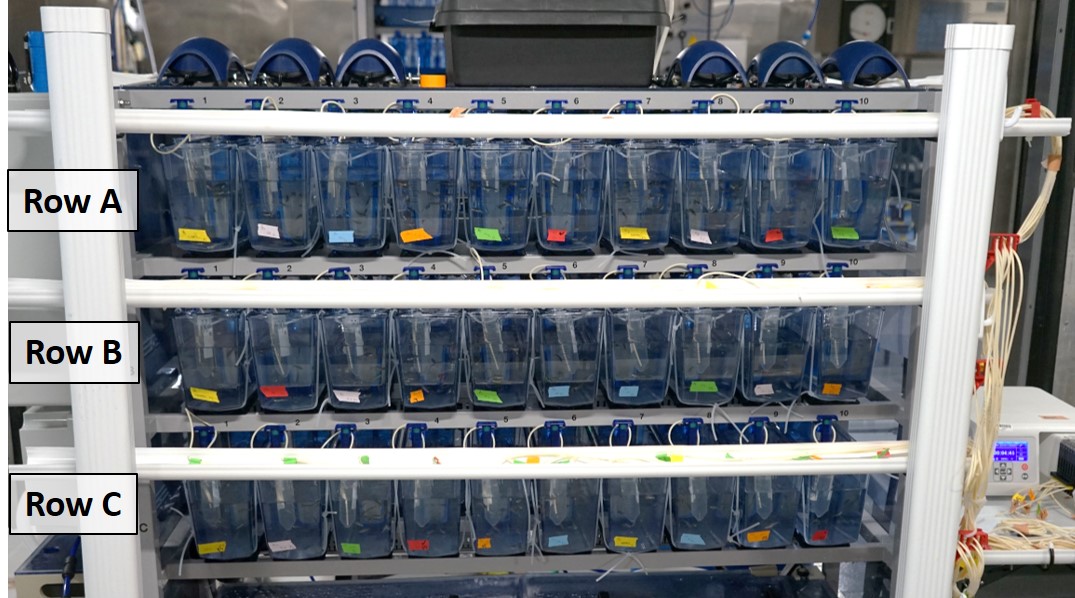


Supplemental Figure S3. Image showing stratified-random placement of experimental treatment replicates (each exposure chamber) on the fish exposure apparatus. Treatments were randomized such that each row included a minimum of one and a maximum of two replicated chambers for each treatment. Tape colors on chamber faces represent concentrations: Yellow = Control; Pink = 0.1 µg/L; Green = 0.6 µg/L; Orange = 3.2 µg/L; Red = 20 µg/L; Blue = 100 µg/L.


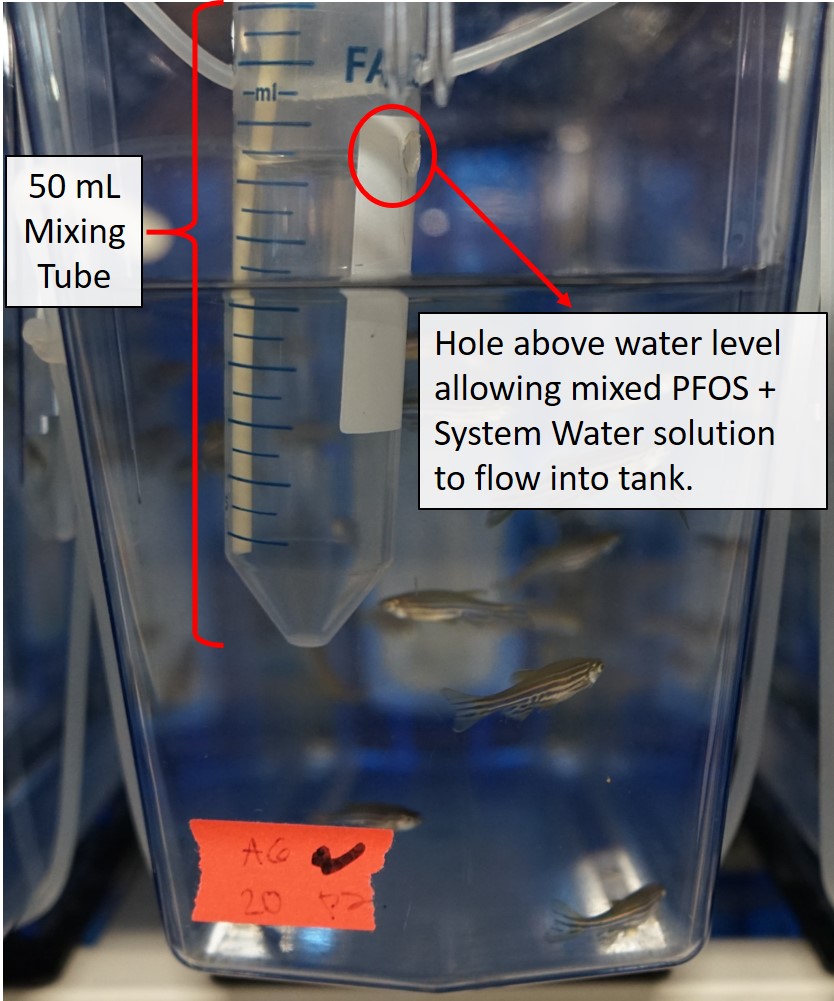


Supplemental Figure S4. Image showing 50 mL mixing tube in exposure chamber containing 20 µg/L PFOS solution. A hole was drilled into the tube to allow mixed 10x PFOS solution and system water to flow into the chamber.

Supplemental Figure S5. Analytical measurement of PFOS concentrations by date in the P1, F1 and F2 exposures. Green dashed lines represent the target PFOS concentration while black dashed lines represent ± 30% of target concentrations. Values represent means of 2 sampling replicates.

Supplemental Figure S6. Body lengths measured in the P, F1, and F2 generations representing the mean and standard deviation (n = 5) of the total fish lengths measured in each replicate (relative to controls). Exposure concentrations represent cumulative measured PFOS concentrations for each time point reported as significant figures based on analytical method detection sensitivity. Asterisks represent statistically significant differences (p < 0.05) relative to controls.
